# When the Place Matters: Moving the Classroom Into a Museum to Re-design a Public Space

**DOI:** 10.3389/fpsyg.2020.00943

**Published:** 2020-06-03

**Authors:** Giovanna Barzanò, Francesca Amenduni, Giancarlo Cutello, Maria Lissoni, Cecilia Pecorelli, Rossana Quarta, Lorenzo Raffio, Claudia Regazzini, Elena Zacchilli, Maria Beatrice Ligorio

**Affiliations:** ^1^Department of Curriculum and Evaluation, Ministry of Education, University and Research, Rome, Italy; ^2^Department of Educational Sciences, Roma Tre University, Rome, Italy; ^3^Academy of Arts and New Technologies, Rome, Italy; ^4^Scuola Secondaria di I Grado Statale Norberto Bobbio, Turin, Italy; ^5^Azienda Speciale Palaexpo, Rome, Italy; ^6^Tony Blair Institute for Global Change, London, United Kingdom; ^7^Luigi Settembrini Comprehensive Institute, Rome, Italy; ^8^Niccolò Machiavelli State High School Florence, Rome, Italy; ^9^Department of Educational Sciences, Psychology, Communication, University of Bari Aldo Moro, Bari, Italy

**Keywords:** sociomateriality, urban space, global citizenship education, dialogue, sustainable development goals, Trialogical Learning Approach

## Abstract

In this case-report we describe an experience where alternative places – rather than the classroom – are exploited to implement learning processes. We maintain that this experience is a good example of materiality because it focuses on a project where students had the opportunity to re-design a public space. To this aim, various objects and tools are used to support discussions and exchanges with new stakeholders. Our theoretical vision combines Piaget’s and Vygotsky’s tradition with an innovative framework called the Trialogical Learning Approach (TLA). From such theoretical background an idea of materiality emerges, that refers to material in combination with the social relationships developed around the material. Our case-report concerns a participatory project run by Rete Dialogues, a national school network focusing on global citizenship education. Our research question is: how can this project highlight the connection between the TLA and socio-materiality? Since 2017, around 200 students (age 7–16) and 20 teachers from different schools have been engaged in tackling the degradation of an important square in Rome. The project – “Dialogues in the Square” (DiS) was developed with several stakeholders that contributed to the understanding of critical issues influencing the maintenance of the square, in the perspective of planning, and possibly implementing improvements proposed by students. Crucial is the cooperation with two important urban art projects: (i) the pilot-project MACRO-ASILO, run by the MACRO museum in Rome and aimed at connecting the world of art with the city life; (ii) the “building sites” of the Rome Rebirth Forum, inspired by the world-known artist Michelangelo Pistoletto’s “third paradise” methodology, that encourages responsibility and action taking on sustainability through art. Drawing on data collected through direct observations and video recordings, we aim to show and make sense of the connection between the TLA and socio-materiality, highlighting three key elements: the flexible use of mediation tools, the overcoming of the dichotomy between individual and collective learning through reflection, and the re-shaping of social practices.

## Introduction

The way students learn is still attracting theoretical and practical attention. New definitions of learning and teaching are sought. Academics and experts are now focusing their research on several dimensions previously neglected or misunderstood, such as creativity, collaboration, action competency, communication competency, and space–time relevance (Bellanca, 2010; Hallgarten et al., 2015; Kober, 2015; Nielsen, 2015; Ritella et al., 2016). Traditional learning does not appear to be able to target these dimensions; therefore, a fresh look at educational practices is needed. After having discussed the theoretical underpinnings of our approach, this paper describes a project where materiality is introduced as the empowering dimension that supports the transaction between different learning contexts. We focus on some aspects of the learning processes that have occurred in one of the sessions within our project. Our intent is to make sense of the impact of materiality from two complementary perspectives: the materiality of the learning object (a square in Rome, Piazza Annibaliano) and the materiality of the working environment (a particular room in a modern art museum in Rome, the “words room,” set up for the MACRO-ASILO project).

## Theoretical Background

Where do children learn about the world? How do students form their own ideas? The literature offers a number of answers to these questions, determining both the theoretical vision of how cognition works and the ideal practical setting for effective learning processes. For decades, theories about these topics have assumed the form of a contraposition/polarity between a Piagetian-based and a Vygotskian-based approach.

According to Piaget, knowledge resides in objects, and children retrieve information by manipulating them (Piaget and Inhelder, 1967). It is by querying the elements composing the context in which children are immersed and by making hypotheses about how objects will react to actions performed “on” them that they gather information about the world (Spelke, 1991), whereas for Vygotskij (1978), the main source of learning is social interaction. It is by observing and imitating adults and, later, by engaging collaboratively in joint actions that children learn and make sense of the world around them. Objects are important, but they become sources of information through social interactions, first based on adult imitation and later by appropriating and internalizing the actions observed.

An attempt to reconcile these two approaches into a wider vision has been offered by frameworks such as situated learning (Anderson et al., 1996; Cobb and Bowers, 1999), Activity Theory (Engeström, 1999), socio-constructivism and cultural psychology (Bruner, 1996; Cole, 1998), and most recently, the Trialogical Learning Approach (TLA) (Paavola and Hakkarainen, 2014; Sansone et al., 2016).

All these approaches share the idea of learning as a complex process that interests the individual sphere, as well as group work, and is influenced by the context and the instruments/tools used. In particular, the TLA integrates three different perspectives: (i) a “monologic” vision of learning, focused on individual increments of knowledge; (ii) the dialogic viewpoint that stresses the relevance of dialogue and encounters of different perspectives; and (iii) the intentional processes involved in the production of collaborative artifacts, connoted by a real meaning and utility. This approach responds to the demands of training competences for the twenty-first century, such as the skill to work with knowledge and to contribute actively to the development of modern society (Karlgren et al., 2020). Furthermore, it capitalizes on insights coming from the socio-constructivism and the cultural approach by giving relevance to context and situated dynamics.

The TLA calls for the construction of the so-called trialogical objects. These objects are addressed to a community that is different from the one in which they are built. To have recipients from another community gives sense to confrontation, contamination of practices, and ways of thinking. Therefore, learners become professionals of knowledge building, capable of creating valuable material objects containing knowledge, which can then be exploited outside school or academic contexts. When objects are used in concrete situations, they create further knowledge through processes of confrontation, generation of ideas, and creativity. Learning becomes a strategy to solve emerging problems and to constantly seek new and innovative ideas. Environments intentionally designed for knowledge innovation, equipped with technological tools, are needed to transform students’ intangible ideas into digital entities (Hakkarainen, 2009).

Within the traditional TLA framework, materiality is still underdeveloped. The focus on building objects that embody conceptual knowledge and shared ideas and the relevance of tools as instruments that foster cognitive and social processes and support the construction of objects are hints of an implicit materiality or rather socio-materiality. Illuminating is Latour’s (2005), challenge (2005) when he asks the reader to define a soldier. Through this simple thought experiment, he concludes that there are no soldiers without their uniforms and arms. They co-constitute each other and determine their relationship by identifying the formation they belong to.

Sørensen (2009) uses the term *materiality* to refer to both the material and the social relationships developed around the material. This definition is particularly helpful when objects are digital. Johri (2011) proposes “socio-materiality as a key theoretical perspective that can be leveraged to advance research, design and use of learning technologies in the practice tradition” (p. 210). The use of technology makes it clear that learning is both inherently material and social or socio-material (Orlikowski and Scott, 2008). When talking about digital environments and tools, the inseparability of material and social elements is essential (Barad, 2003; Barzanò and Grimaldi, 2013).

The theoretical concept of materiality is operationalized in different pedagogically oriented strategies such as Object-Based Learning. For example, Mayorga (2019) reports that while handling museum Objects, primary school students start to think differently and to reinterpret the cultural artifacts. Mirza (2016) observes that the material dimension assumes the function of a medium through which primary school children project their own emotions or those of another person or convey information and contribute to knowledge construction.

Thus, materiality is not just a matter of adding a new dimension; it means highlighting the relevance of considering “things” as real partners of cognitive and social processes, as elements containing knowledge and supporting the generation of new knowledge. This knowledge is not simply acquired by touching, manipulating, or experimenting with “things”; rather, it is defined through social actions, cultural processes of sense-making, and intersubjective construction of mutual exchange of values about the objects. Where and with what this is occurring matters because it contributes to shaping these processes.

## The Case Study

The case study presented here aims at providing empirical evidence of the role of socio-materiality in shaping learning processes. We also highlight how the TLA helps to emphasize the socio-material dimension, crossing the boundaries between formal (classroom) and informal (museum, the square) learning spaces. This will allow us to answer our research question: how can this project highlight the connection between the TLA and socio-materiality?

The session we analyze has been developed within a project called Dialogues in the Square (DiS). Started in 2017 – and still active – it has involved over 200 students from primary school (age 7) to upper-secondary school (age 16) and 20 teachers, in two schools situated in central Rome: Istituto Comprensivo Settembrini and Liceo Machiavelli. Within a framework of activities targeting global citizenship education (Reimers, 2009; Sobe, 2012; Reimers et al., 2016) and sustainable development goals run by a national school network (retedialogues.it), students started brainstorming about their environment, focusing on the needs of a nearby well-known square in Rome: Piazza Annibaliano. This important space, recently restored (2014), was soon left in a dangerous abandonment. A new metro station, situated in a context of ancient monuments, is now surrounded by litter and unfinished flowerbeds, left uncultivated. Students were encouraged to observe the square and engage in planning its regeneration: their plans are conceived as trialogical objects, i.e., knowledge that they create addressing communities external to the school. Moreover, negotiations were started with the municipality to have their support, resulting in a formal memorandum of understanding (MOU) with the schools. Artists/experts in various fields were involved to help students figure out suitable actions to undertake to improve the state of the square, eliciting its potential as a social and artistic site.

In 2019, an important opportunity arose: a well-known museum of modern art – MACRO, not far from the schools and the square – launched its pilot project MACRO-ASILO aimed at promoting the connection between citizens and art and making its spaces available to artists or citizens with ideas to present. In particular, the MACRO’s “words room” appeared to be the ideal venue to work on the DiS project. This is a classroom-style room equipped with rolling chairs and tables and with an enormous traditional blackboard, measuring 22 × 3 m. The museum also became the venue of the Rome Rebirth Forum, an ongoing initiative promoted by the world-renowned artist Michelangelo Pistoletto to enact his idea of the “third paradise,”^1^ involving artists and social actors to develop and spread a deeper awareness on sustainability issues. The DiS project became an active member of the forum and benefited from the opportunity to invite several artists to cooperate.

Several sessions took place in the “words room,” where different classes worked with/on the blackboard to accomplish “planning activities” concerning Piazza Annibaliano. Students sketched their proposals after lively discussions with artists/experts. Each session was public, had a title, was scheduled ahead, and was published in the museum’s catalog: invited guests and occasional visitors were welcome, allowing students to share and discuss their performance with various audiences (see a detailed visual presentation of the full project in the Supplementary Material).

In the next paragraph, we will describe the setting, the available equipment, and how tools became partners of students’ cognitive and social processes.

### A New Learning Space: Getting Into the MACRO Museum “Words Room”

In this section, we analyze one particular event taking place in the MACRO museum’s “words room,” focusing on the learning environment, the materials used, and their impact on participants’ reactions and interactions undertaking the task. In this session, a single class is involved, composed of 27 pupils aged 12 (grade seven, 15 girls, 12 boys) from mixed socio-economic backgrounds. They are familiar with the square, as they all live nearby. The class is very active within the DiS project; nevertheless, it is their first time in the MACRO museum. The session is observed and videotaped: our data consist of extracts from students’ dialogues as well as “thick descriptions” (Denzin, 2001) elaborated by the external observer.

**FIGURE 1:**
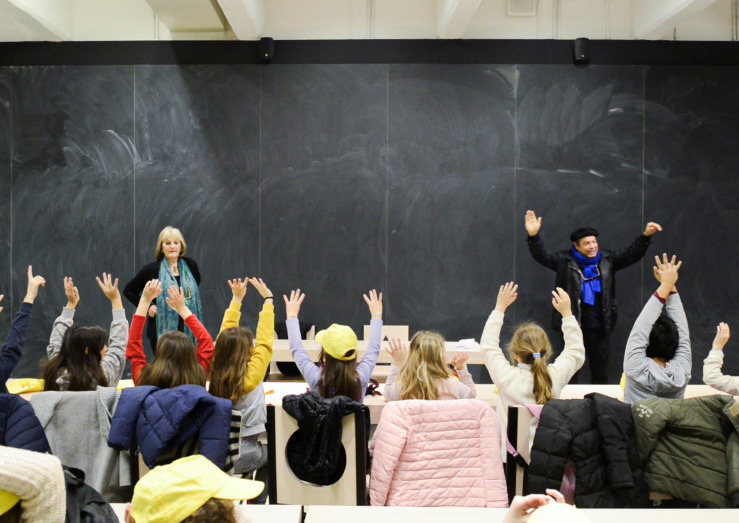
Film director Rachid Benhadj introducing the exercise to the students. Images © Martina Pavia, graduating student at Academy of Arts and New Technologies, Rome, Italy.

It is 7 February 2019, from 10.30 to 13.30, when our class goes to the museum with their art and technology teachers to meet Rachid Benhadj, a leading Italo-Algerian film director particularly interested in diversity and intercultural dialogue (see Figure 1). The students know him, having watched one of his videos. As is the case for artists/experts in other sessions, he was invited to support students’ creative process of elaborating the idea of the “square” as a venue for proposals and new atmospheres that can add value and expand the possibilities of inhabitants and visitors.

In a preliminary meeting in the museum hall, five teams (four or five students each) are formed, following the teachers’ suggestions. Benhadj presents his proposal to the students: “Think deeply of Piazza Annibaliano, figure out new settings, and portray them following the wave of your dreams: how would you like the square and why, pushing your imagination as far as possible…” Students are, therefore, invited to elaborate the idea of the “square” representing their ideals, without worrying about feasibility at this stage. With this task in mind, they enter the “words room,” and it is clear how impressed they are from the beginning by its lights, the arrangement of the rolling furniture, and the giant blackboard. A connection between thinking/doing is thus made evident, and students are encouraged to go back and forward from immateriality to materiality – as we will see in the next paragraph.

### At Work: Flexible Use of Mediation Tools

Benhadj sketches a quick map of Piazza Annibaliano and surrounding streets at the center of the blackboard and better clarifies the expected delivery: paper-and-pencil sketches to start, and then the teams will move to the blackboard to represent their project with colored chalks.

**FIGURE 2:**
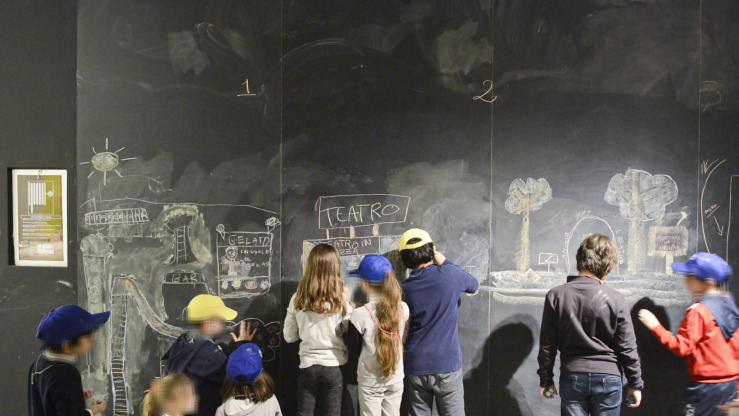
Students sketch their ideas for the square. Images © Martina Pavia, graduating student at Academy of Arts and New Technologies, Rome, Italy.

Now that the task is clear, students start working on white sheets. Talking becomes intense, ideas are shared, and sketches circulate within/between teams. Technology comes into play naturally; no need for adults to suggest it. For instance, phones become cameras to store pictures that make possible comparisons and overviews crucial to inspire the work on the blackboard. Finally, about 45 min after starting, the five teams position themselves around the map sketched by Benhadj, easily defining their action space on the blackboard (see Figure 2).

The “genius loci” of the room lies in the alteration of the dimensions of traditional tools used in the classroom. This setting ends up disregarding a consolidated stereotype: the blackboard is by definition an “exclusive” place generating a markedly vertical relationship. It is used by a single person – or a few – who is expected to report something to an audience to whom the back is turned. Here the blackboard is “open to all”: the teams work horizontally and simultaneously, observing one another’s work and sharing ideas. Apparently, the confusion is remarkable, but the works develop efficiently; students’ active engagement is visible. Someone moves his or her chair near the blackboard, others use the ladder available in the room, and someone else even sits on the shoulders of a friend to use the space at the top of the board. Others shoot videos or take pictures.

**FIGURE 3:**
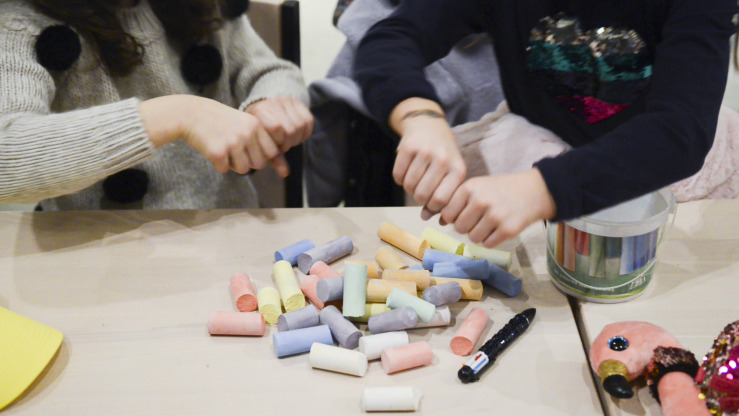
Chalks acted as an enabler of creativity. Images © Martina Pavia, graduating student at Academy of Arts and New Technologies, Rome, Italy.

Even the colored chalks become important actors, with their immediate but fragile effectiveness enabling creativity (see Figure 3). Paradoxically, the awareness that whatever was created can disappear with just a few passes of the eraser pushes students to refine their work: “to take pictures before it disappears,” as a student clarifies.

What has been described so far provides first evidence of how the TLA could enhance the socio-material dimension of learning. This approach emphasizes the flexible use of technologies and mediation tools. Depending on what students want to achieve – create, store, transform – they move from using their smartphones to using chalks, always as a tool to shape their ideas and to “materialize” them.

### Reflecting on the Work

**FIGURE 4:**
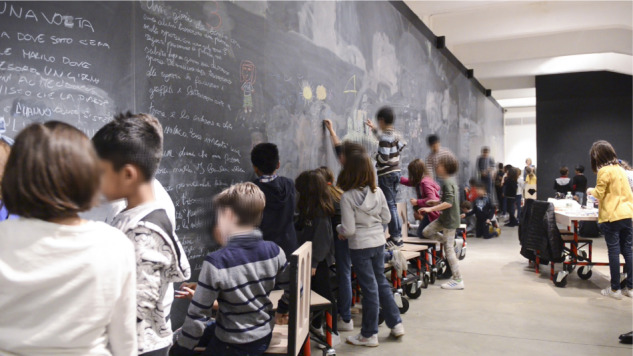
Students using the full length and height of the blackboard. Images © Martina Pavia, graduating student at Academy of Arts and New Technologies, Rome, Italy.

In about one hour, the blackboard is lively, full of shapes, colors, and writings, and the time comes for a collective report (see Figure 4). Benhadj poses two questions: “What have you done, can you tell us?” And then: “Were there emotions in this work? What touched you the most?” Each team gets ready for their presentation, while someone enjoys looking at their work from a distance, video-recording a full overview of the blackboard. The teams “walk” along the blackboard, stopping in front of each drawing to deliver the presentations: students naturally swing from the role of presenters to that of audience. Feedback is intense.

Proposals are detailed, rich in inventions and strategies. They include: architectural and decorative elements, green spaces, and many solutions about how to make them work. Director Benhadj is very pleased; he listens carefully and interacts with students, to their great satisfaction. For example, project 3 presents a wall specifically created to welcome graffiti artists. Next to the sketch, some guidelines appear on how to organize periodical cleaning, to allow for writers’ rotation. In project 4, the main attraction is an artificial tree, a sort of sculpture, with a central clock and four branches, each colored with seasonal vegetation, indicating four different paths corresponding to the seasons and their emotional atmospheres. Luca^2^ – a student from team 4 – explains: “If you feel sad, maybe for a bad mark at school, you can walk the winter path; but if you are happy, you go for the spring one!”

When the time comes to answer director Benhadj’s second question about emotions and surprises, excitement increases: nobody wants to give up telling their experiences. Keywords in the narratives are: expectations, satisfaction, freedom, and team work. Several students underline how they did not expect to experience such intense satisfaction in working together. Pointing to their drawing, visibly excited, Carla from team 3, claims: “I didn’t imagine we could do something like this… now I see it! I think it’s very original.”

The blackboard with its significant size has made everyone’s work visible in real time: a multiplier of satisfaction, creating opportunities for feedback, expanding the meaning of “audience.” The idea of satisfaction is expressed by students in many ways: “to see what you just did and realize that everybody looks at it” (Luisa), “to know that before there was nothing and now… look here!” (Angela), “to understand that maybe we will be able to change something with our drawings” (Oscar), “to work so freely in cooperation and share the product” (Eleonora).

More than just simple satisfaction for the work done emerges here. Students overcome the dichotomy between individual and collective approaches to learning, clearly showing the contribution of the TLA to socio-materiality. Productive participation in knowledge creation processes needs the transformation of personal contributions toward the construction of collective products that “embody” the shared enterprise. Our students are involved in such creative processes; therefore, their individual contributions are intertwined in social processes.

### A Critical Incident: Re-shaping Social Practices

In the “words room” session, several “critical incidents” occurred, in the sense indicated by Tripp (1993, 2006): events that produce new interpretations and allow their significance to be unraveled. We focus on an emblematic example: the case of Marco, a clever but difficult student from team 2. When students are invited to stop drawing, Marco furtively takes a chalk, quickly sketches a little circle under his team’s drawing, and writes something inside it, confusedly. He looks around with a somewhat guilty expression, almost waiting for a reproach for not putting aside the chalk. One of the teachers asks him: “What were you in such a hurry to write?” Surprised by this attention, lacking any punitive intention, he replies: “I wrote: *this is for you from us*.”

Marco feels entitled to act, breaking the order given (putting aside the chalk), probably because of the new setting. The large blackboard is a material space inviting to be filled. Even the teacher reacts in an unexpected way: she asks for the reason of such behavior instead of reproaching Marco. The setting elicited new social practices from both the teacher and the student, allowing the discovery of Marco’s awareness of having achieved something that deserves to be offered to others. Both teachers are astonished at the involvement transpiring from the words of this challenging student.

The TLA posits that by solving complex, “authentic,” and challenging problems, social practices are re-negotiated based on the contamination offered by entering new settings and using flexible tools. This is exactly what happened in our case. This experience created the space for new ways of interacting, for both teachers and students. Crossing boundaries between settings – school and museum – represents a crucial experience to review the practices supporting the creation of objects, such as how to react when a student does not follow the teacher’s indications.

## Discussion and Final Remarks

In this research, we have tried to explore how learning and teaching change when located in an alternative place. Our theoretical lens, in particular the TLA approach, allows us to understand the learning context as a triadic relationship between learners, teachers, and objects. Since the relationship between socio-materiality and the TLA needs to be further explored, we have provided some empirical evidence of their connections. Indeed, the MACRO-ASILO’s “words room” has proved to be a rich space, creatively challenging students and putting teachers and students in a novel situation. A typical school setting, which traditionally enhances top–down interactions, has now become a space for all through the huge blackboard, where unexpected processes occur and productivity flourishes, creating an impact on students’ ideational processes and their performance. Students have explored all of its potential, positioning themselves – both physically and cognitively – in different ways to draw, discuss, and observe, making their emotions more alive. As shown elsewhere (Cattaruzza et al., 2019), the space with its objects becomes part of the interactive actions. All participants, including non-traditional school actors – director Benhadj in our case – form a virtuous triangulation, where each element enriches the other. In this sense, the contraposition between Piaget and Vygotskij is overcome: knowledge into the objects and knowledge possessed by human actors compose a complex polyphony, made by many types of “voices” and different rhythms (Bakhtin, 1981).

Even research conducted in non-school contexts (Kumpulainen et al., 2014; Rajala and Akkerman, 2019; Yrjönsuuri et al., 2019) has shown how objects participate actively in shaping the learning process. Similarly, we found that students’ engagement improves greatly, and it goes beyond learning concepts so that collaborative and creative knowledge building is possible. When students are challenged to produce useful objects for a large community, they feel part of this community – becoming active citizens – and feel entitled to improve it.

Using a large blackboard and moving furniture, students have had the chance to work together, experiencing their mutual influence and the impact of cooperation in real time, together with a sense of self-efficacy (Bandura, 2010). Learning is now not only connected to the possibility to build knowledge, but it emerges from the deep engagement elicited in the continuous shift from presenters to audience: question–answer processes were intense, and new interpretations of traditional solutions arose, encouraging creative developments. The triangulation learners–teachers/expert–object was activated by the new “place” where objects composing the setting (the blackboard, the chalk, the cameras, and the other technological means) functioned as mediators to build a new common object: the imagined square. Moreover, the meaning of the various dimensions tackled by the project was exploited, and the museum has offered a place where learning means “giving body” to ideas, concepts, and social interactions.

We witnessed how materiality implies also the interconnection between different time–space levels. One level is the local context in which students are working, in our case, the museum. The other levels concern the contexts evoked; one could be the physical square visited and studied by the students and/or the imagined square they were planning. Another level pertains to the classroom, where a large part of the preparatory work was done.

As Säljö (2019) contends, instruments are tools meant not only to build objects but also to think with and through them. So, the target object – the square in our case – becomes an additional material object to reach new cognitive levels where many points of view may interweave. This leads to further levels, which in our case concern the symbolic value attached to the object. These values are constructed through various discourses and representations of the object. The square, therefore, becomes an agora to think, a space to meet, a venue for art, a central hub for business, and a destination and point of departure.

In conclusion, in this experience, learning is a process that is deeply affected by the space and place in which it occurs and by the materials available. Such materiality has a multi-level dimension where each level enriches the other and all together influence the learning outcomes.

## Data Availability Statement

All datasets generated for this study are included in the article/Supplementary Material.

## Ethics Statement

Ethical review and approval was not required for the study on human participants in accordance with the local legislation and institutional requirements. Written informed consent from the participants’ legal guardian/next of kin was not required to participate in this study in accordance with the national legislation and the institutional requirements.

## Author Contributions

The authors have shared the responsibility for the theoretical framework, data collection, documentation, and writing.

## Conflict of Interest

The authors declare that the research was conducted in the absence of any commercial or financial relationships that could be construed as a potential conflict of interest.

## Footnotes

1 http://www.pistoletto.it/eng/crono26.htm.

2 Student names have been changed to protect their privacy.
