# Supplementary material for: When the Place Matters: Moving the Classroom Into a Museum to Re-design a Public Space
Source: Front Psychol. 2020 Jun 3;11:943. doi: 10.3389/fpsyg.2020.00943 (PMC7283608; doi:10.3389/fpsyg.2020.00943)
Supplement: Supplementary file 1 [file Data_Sheet_1.PDF]

Giovanna Barzanò, Giancarlo Cutello, Rossana Quarta, Lorenzo Raffio
Claudia Regazzini, Paola Spallanzani

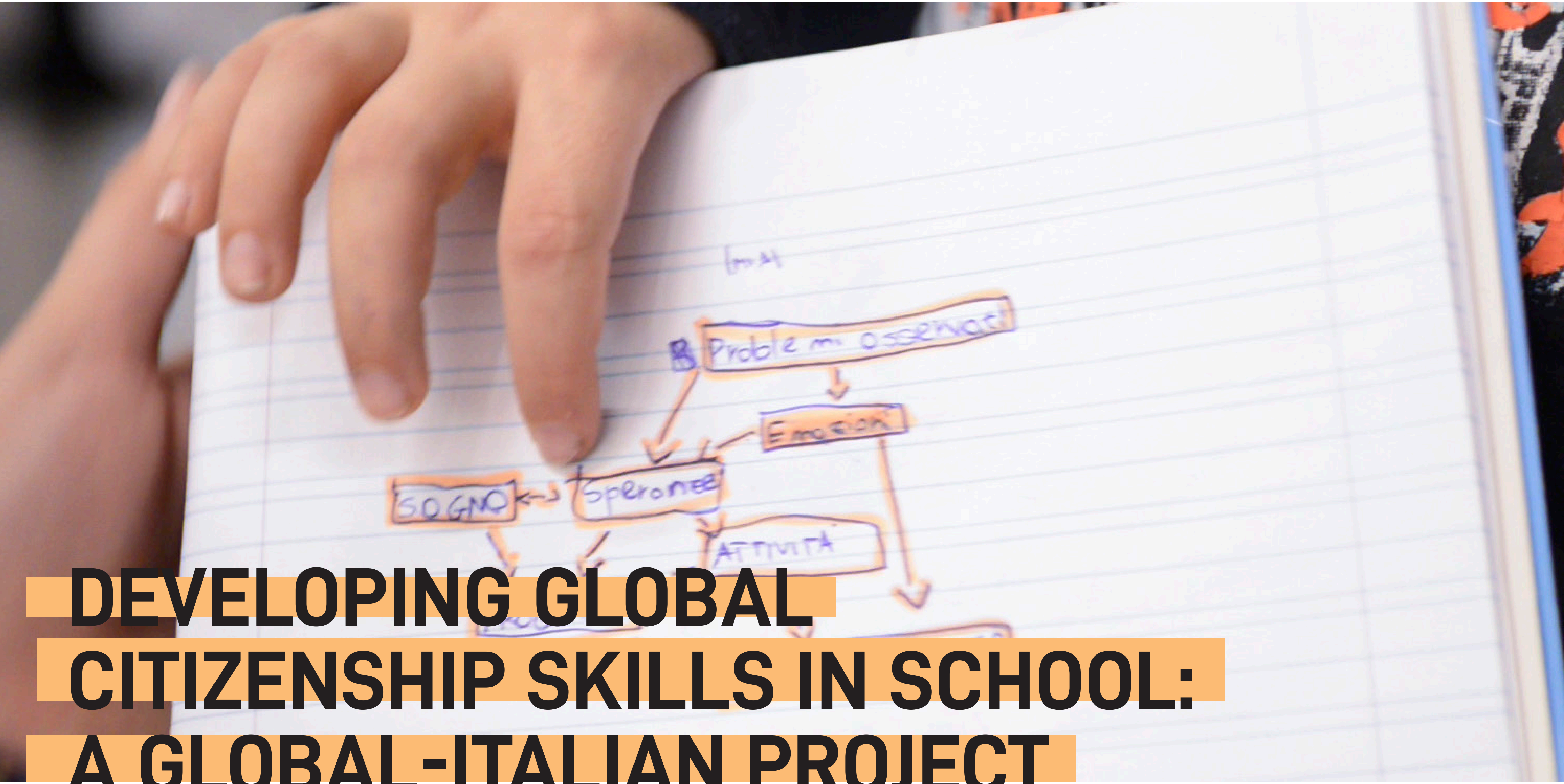

DEVELOPING GLOBAL CITIZENSHIP SKILLS IN SCHOOL: A GLOBAL-ITALIAN PROJECT

Rete Dialogues

GENERATION GLOBAL

ACCADEMIA DELLE ARTI E NUOVE TECNOLOGIE

MACRO ASILO il museo ospitale

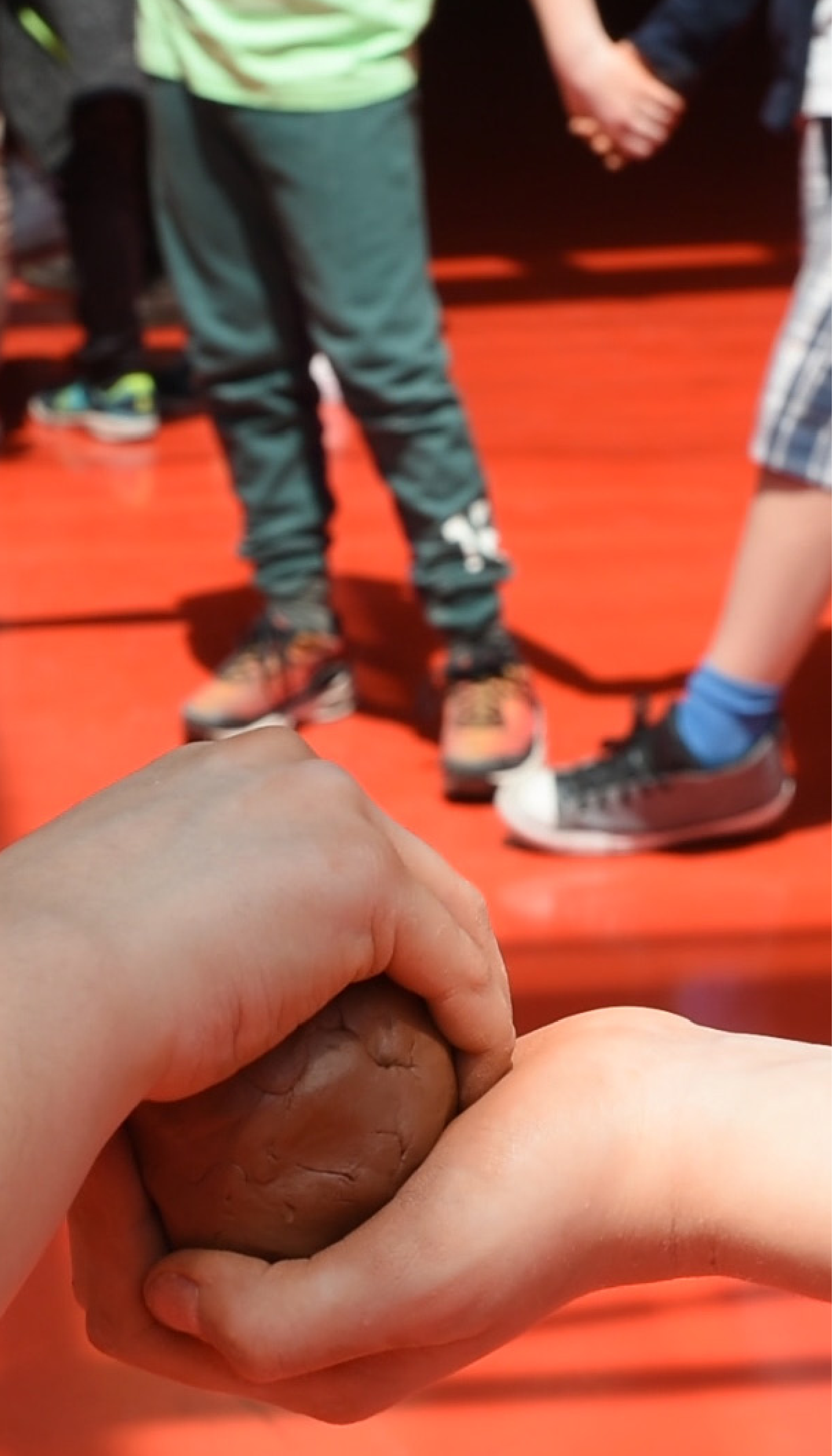

1. THE CONTEXT

The project was born within “Rete Dialogues” and from its relationship with the international project Generation Global, through an MOU between MIUR and the Tony Blair Institute active since 2011.

Rete Dialogues

- is a national network of schools set up in 2012, made up of 30 Primary, Lower Secondary and Upper secondary government schools in different regions

- is an inter-generational professional learning community, where teachers and students learn about the skills of global citizenship through the pedagogy of intercultural and interreligious dialogue

- the network's activities are designed and made available for all the institutions involved

Rete Dialogues

GENERATION GLOBAL

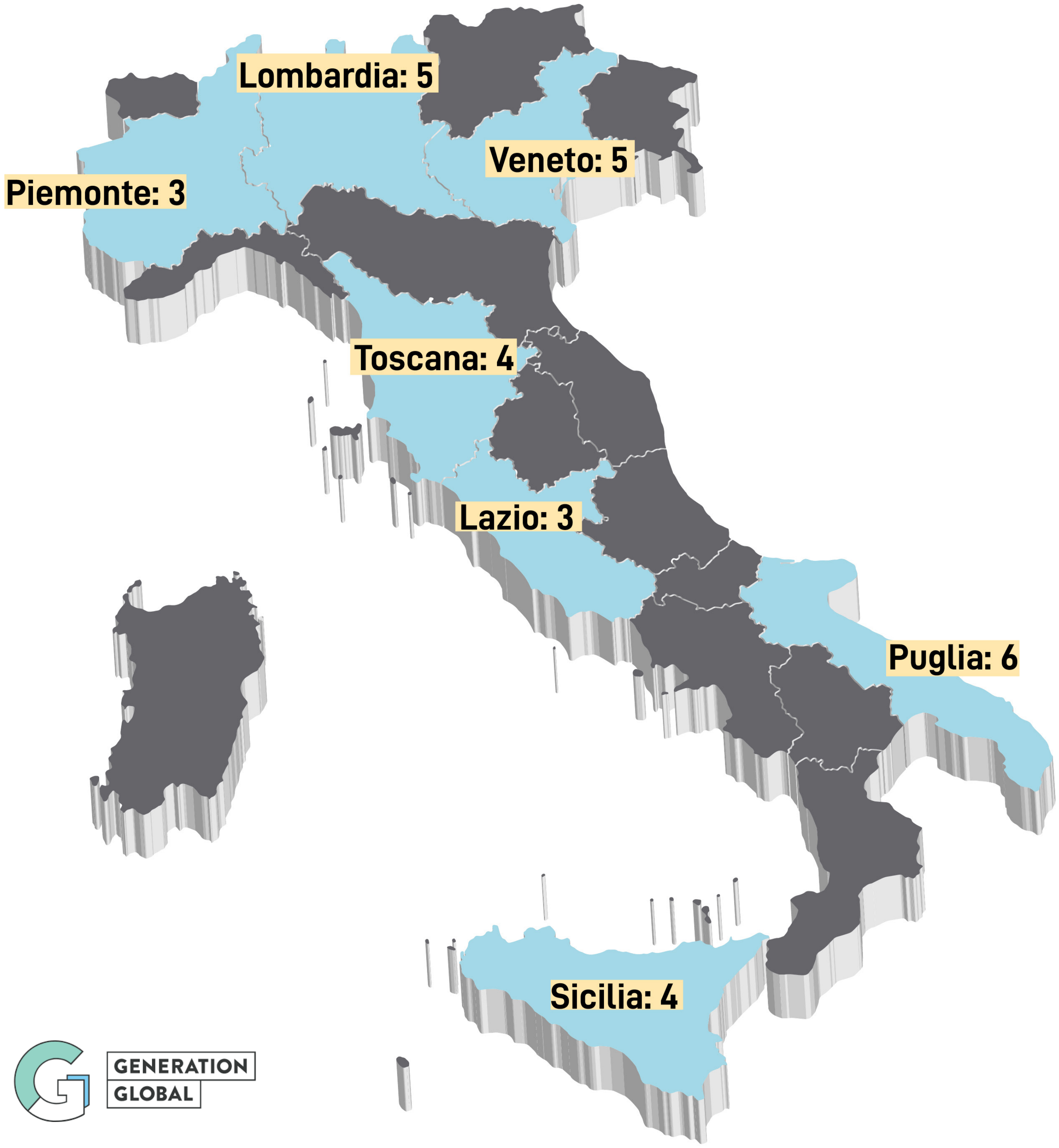

Rete Dialogues

GENERATION GLOBAL

Generation Global

• GenG is an international programme for students aged 12-17, active in more than 20 countries, many of which in high-risk areas

It purports to foster:
the ability to engage in deep and meaningful conversations through the practice of dialogue with students from different cultures and religions in facilitated videoconferences and the participation in a secure online community

Rete Dialogues

GENERATION GLOBAL

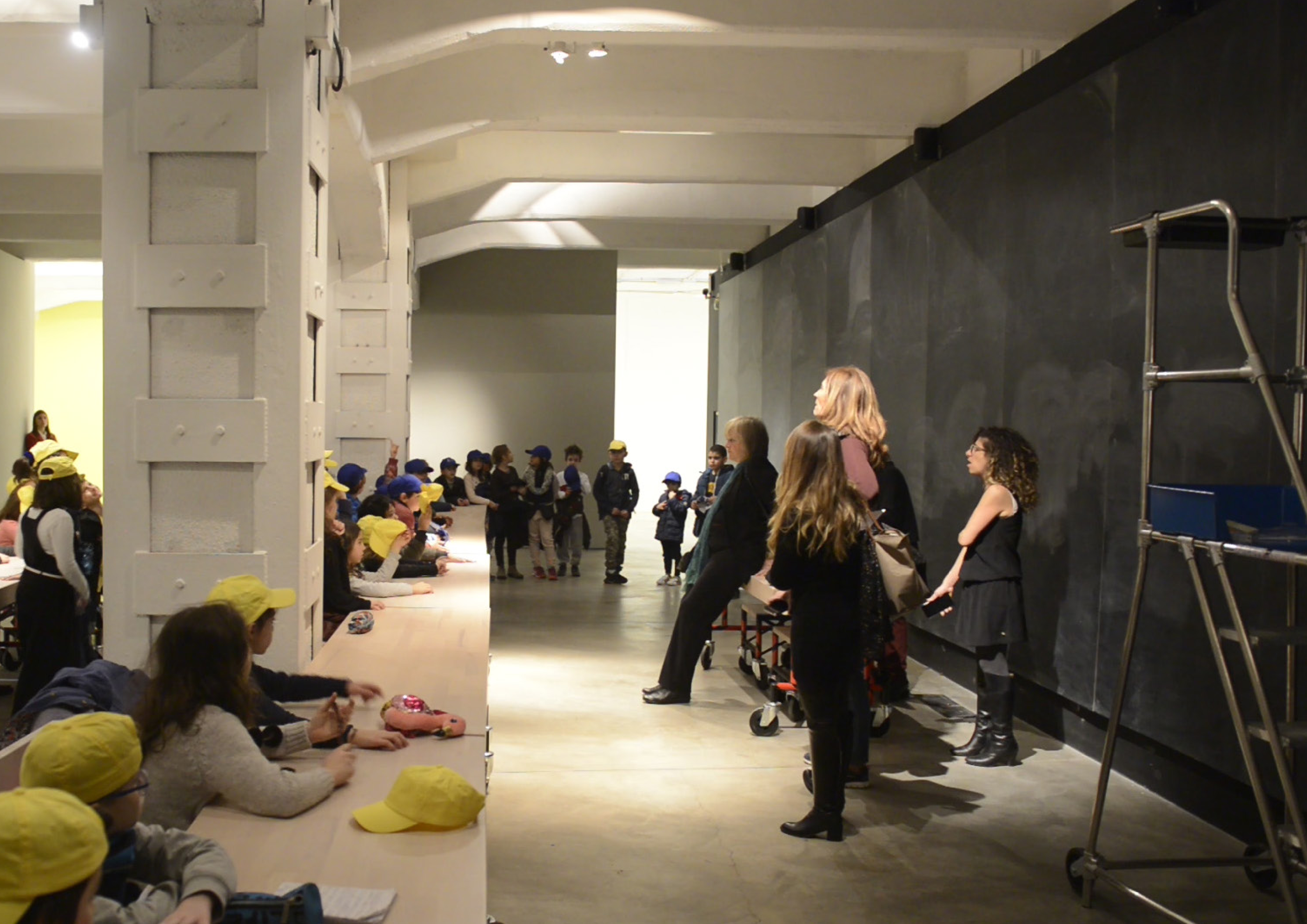

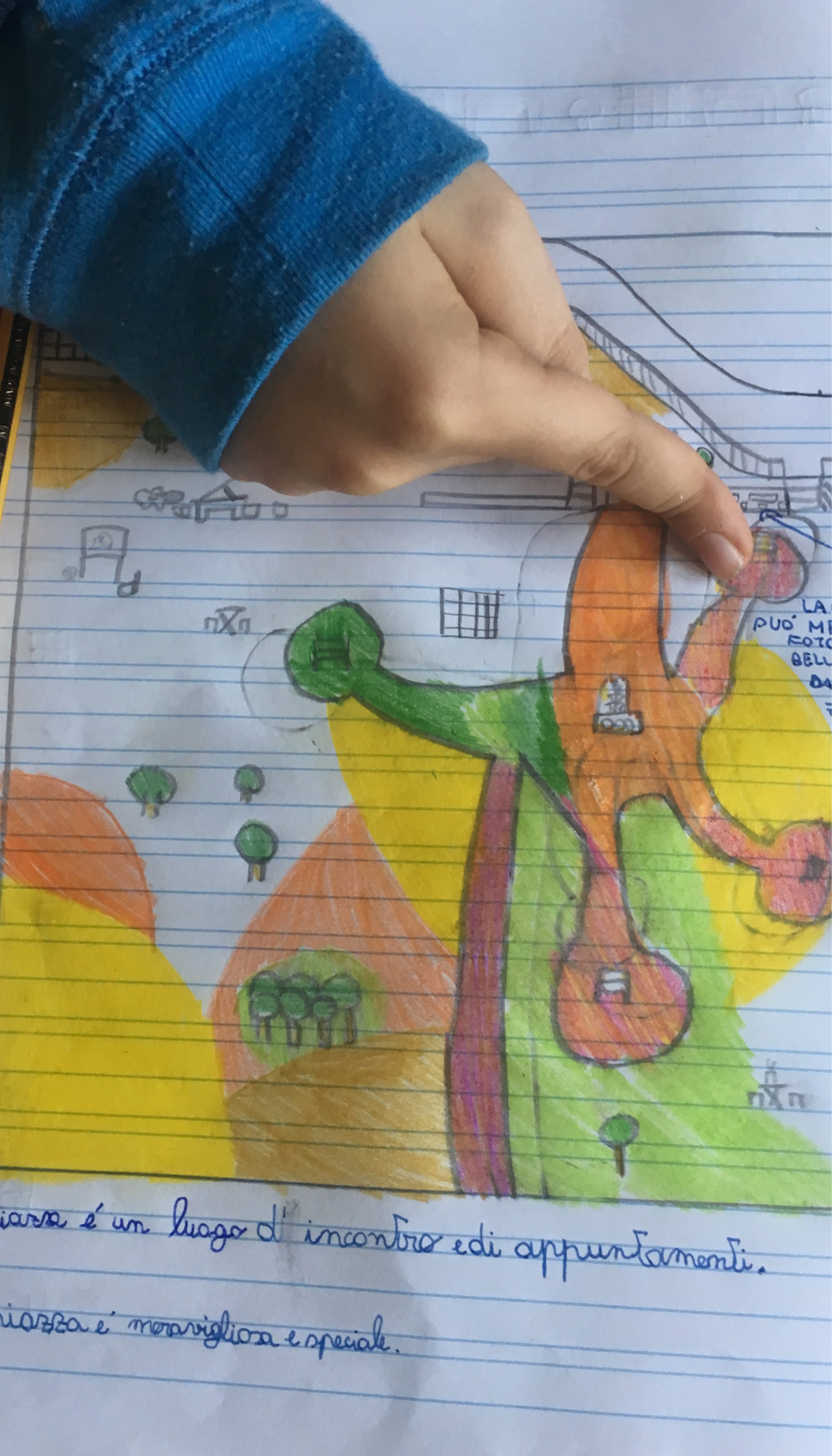

2. EDUCATION FOR GLOBAL CITIZENSHIP

Being a “global citizen” is a complex and multidimensional learning objective, which concerns:

- the ability to understand the most difficult challenges in the world today
- familiarity with communication, media and technology
- awareness of one’s multiple identities and relationships
- one’s positioning in the world
- participation in a solidarity-based sense of responsibility at a global level
- the creation of frameworks for collective initiatives that can generate civic actions to promote positive actions to improve the world.

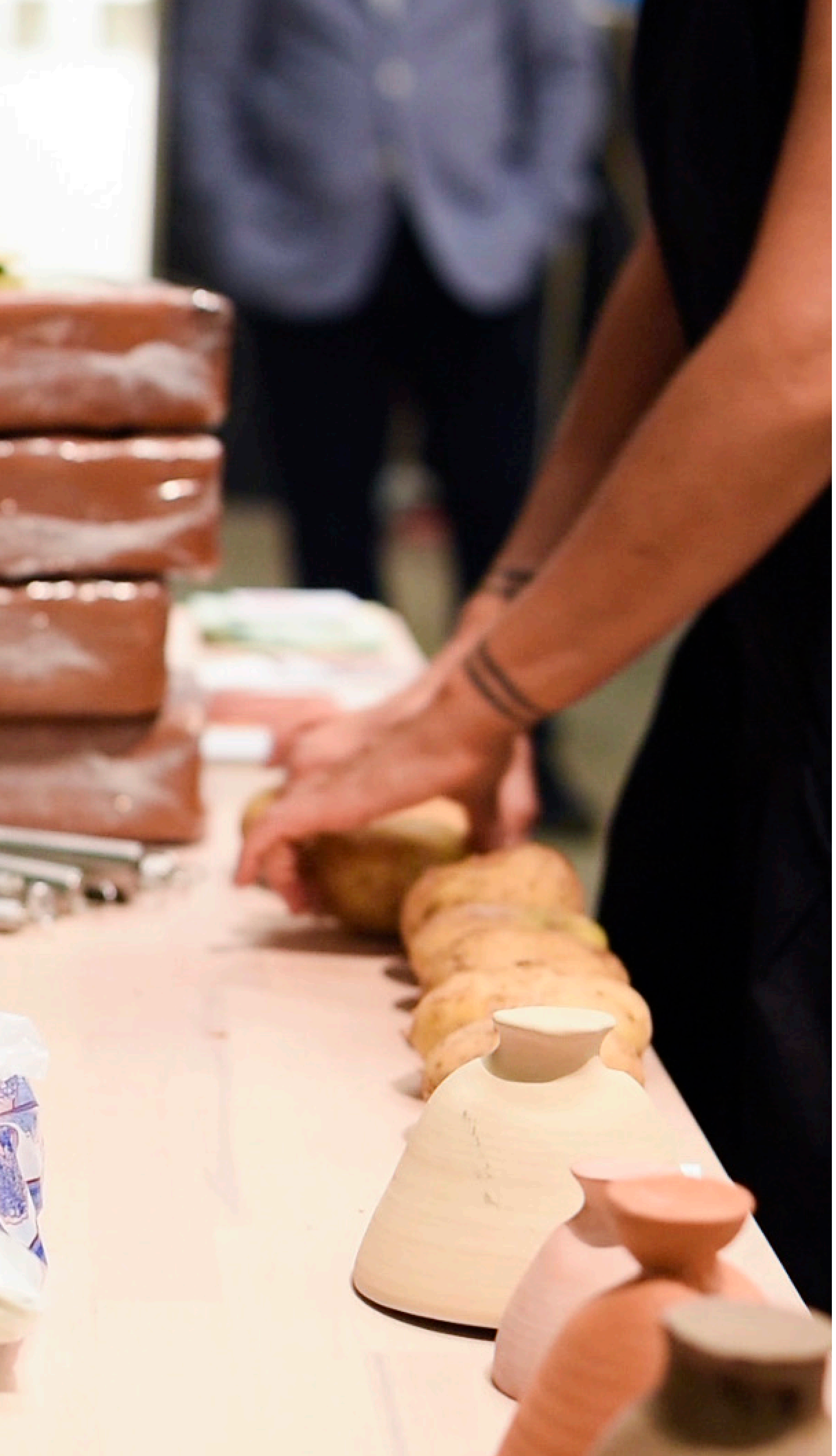

3. IT IS A WAY OF LIVING THE SCHOOL AND ITS SURROUNDINGS

A school that teaches global citizenship must be prepared to find its place in a global world. International literature offers a rich repository of experiences that can highlight the premises:

- **Involvement of the community and of different social actors, dialogue with experts (ASVIS, 2018)**
- **The values of cosmopolitanism discussed and acted out in everyday life (Ritzvi 2009)**
- **“Critical events” in education (Woods, 1993-2012)**
- **Intergenerational learning (Fielding, 2011)**
- **Formal, informal and multimedia learning (Greenhow, Lewin, 2016)**
- **The student as a source of knowledge, co-creator, author, interlocutor (Fielding 2011)**
- **Authentic responsibilities of students with real impact, a sense of belonging to school life (Riley, 2017)**
- **The school is a place for creating new identities (Gee, 2017, Ligorio, 2010)**
- **Group work, collective tasks, peer learning: doing and collaborating (Cesareni, Ligorio, Sansone, 2018)**
- **Participatory research-action activities on perceptions, behaviors, attention to critical incidents (Anderson, 2017)**
- **Teacher professional development through professional learning communities (Stoll, Luis, 2008)**
- **Italian strategy for global citizenship education (Autonomous Province of Trento)**

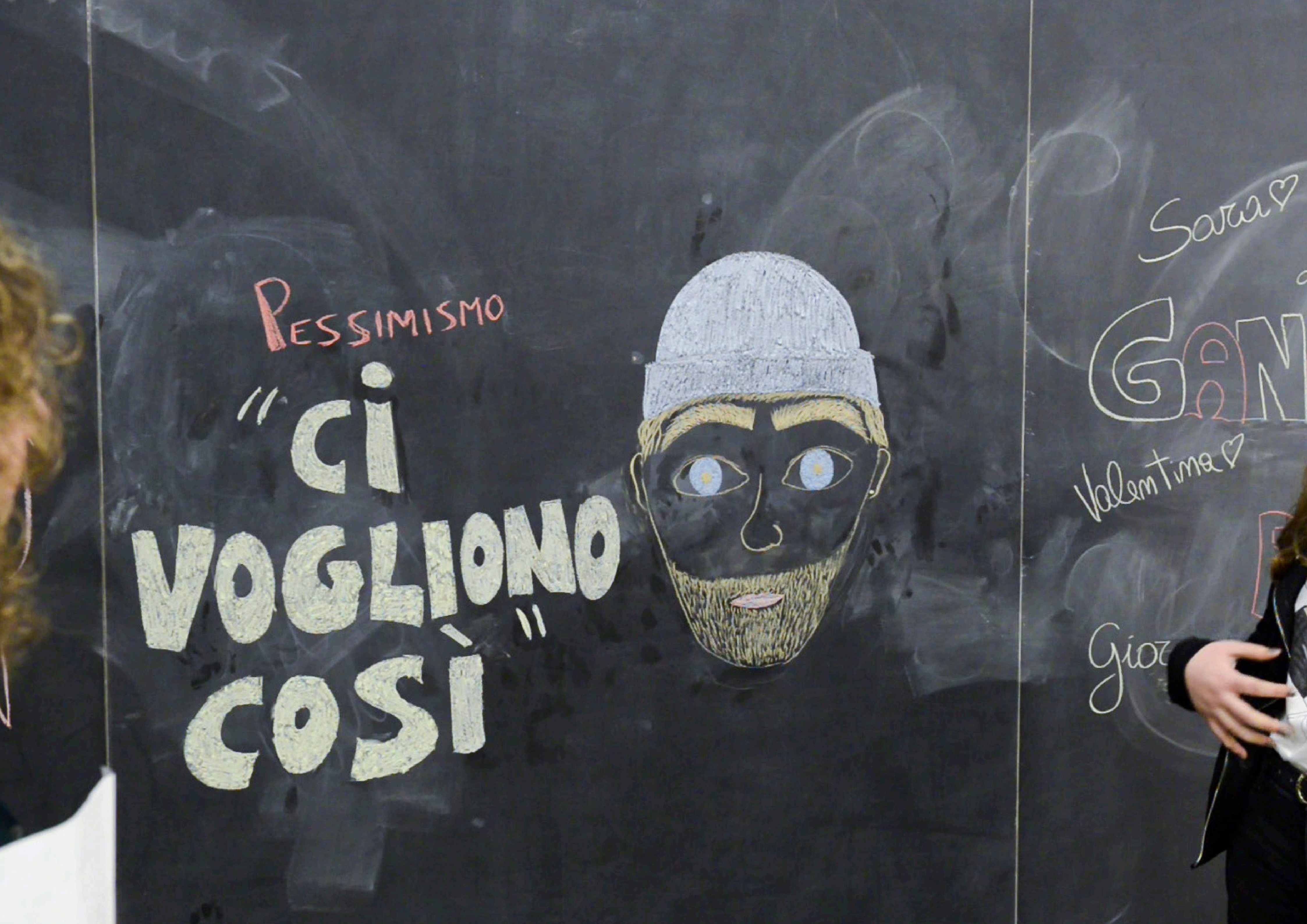
PESSIMISMO
"CI VOGLIONO COSÌ"
Sara♡
Valentina♡

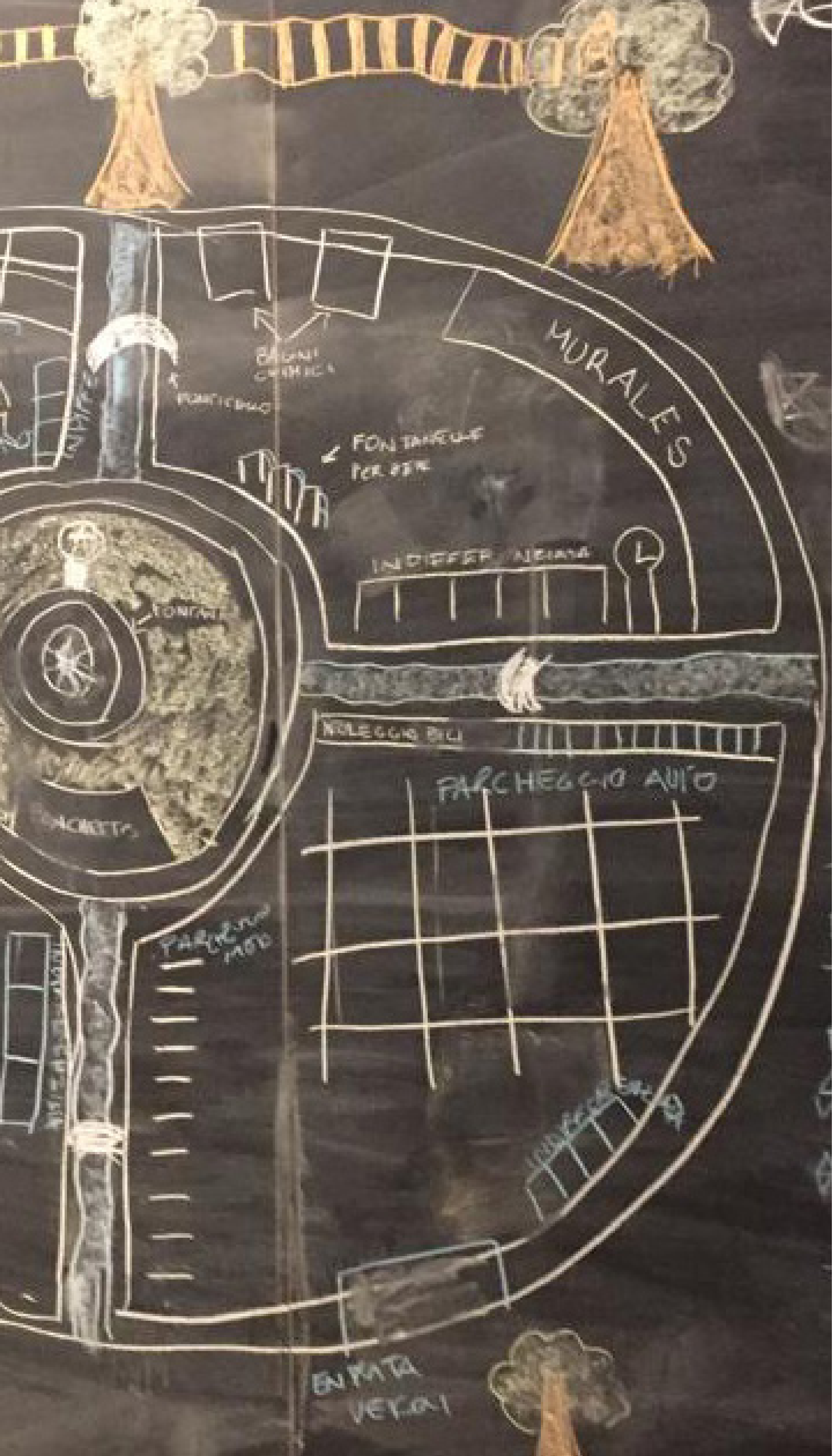

4. FOCUS ON THE 'DIALOGUES IN THE SQUARE' PROJECT AND ITS GOAL

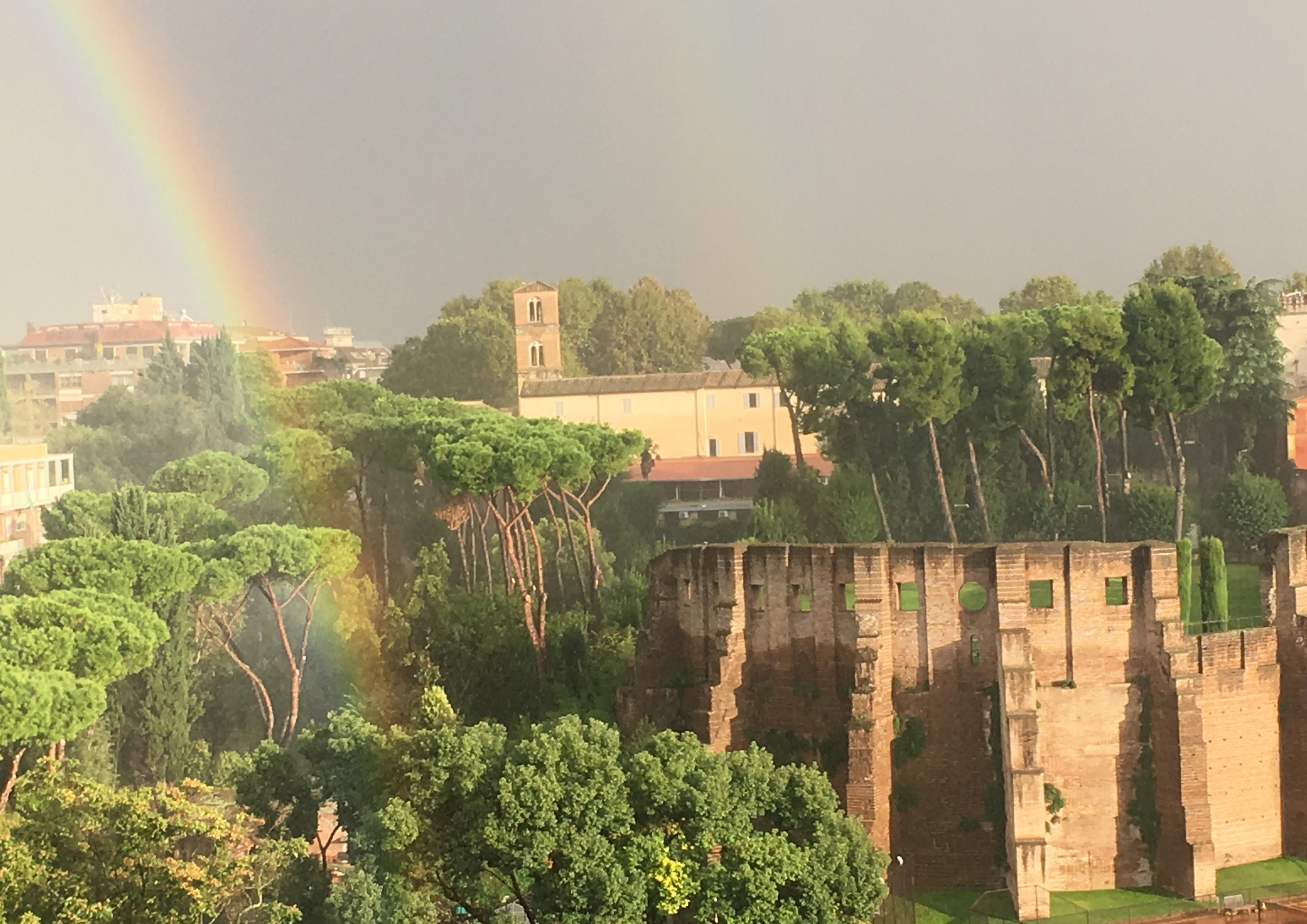

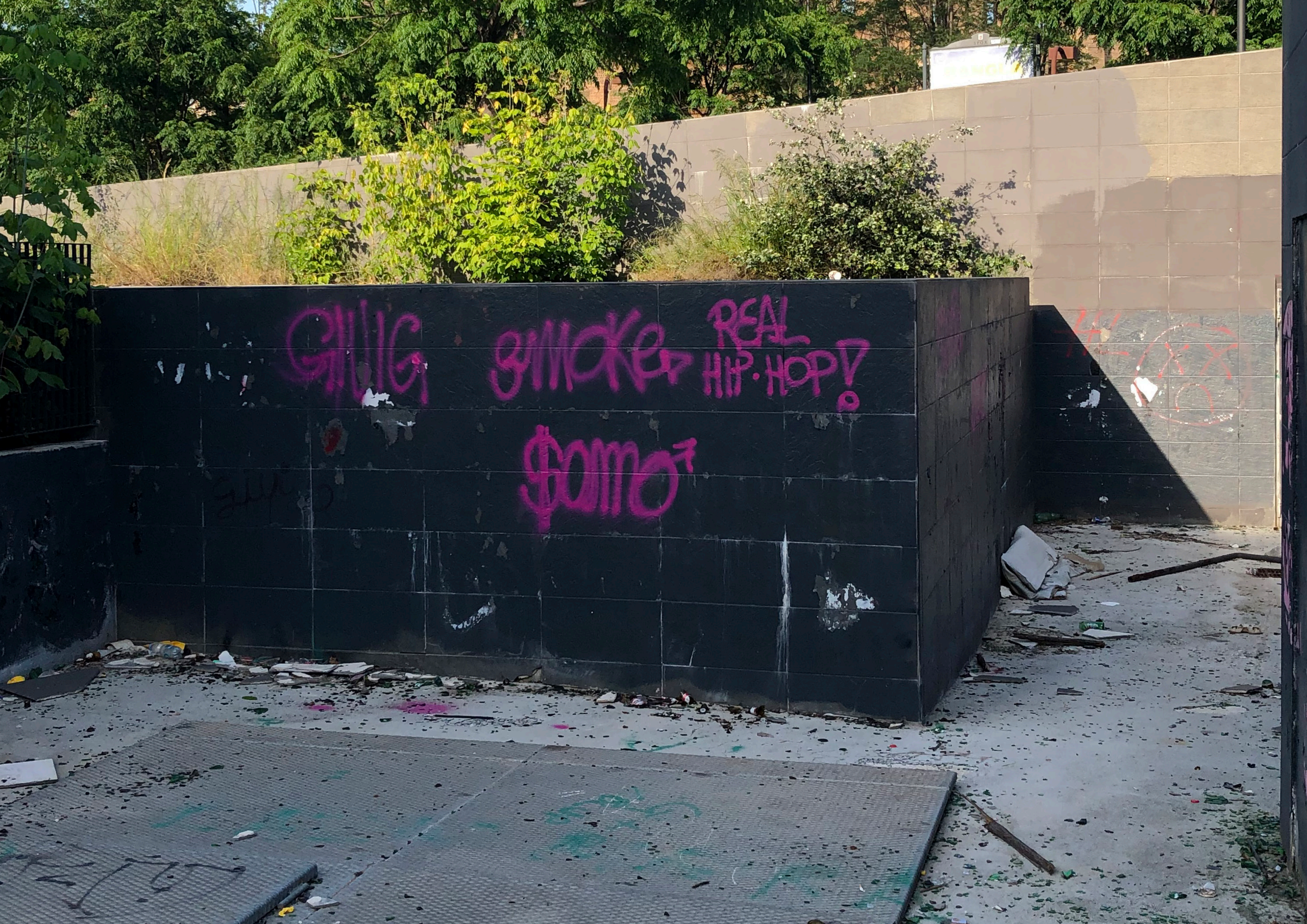
REAL
HIP·HOP

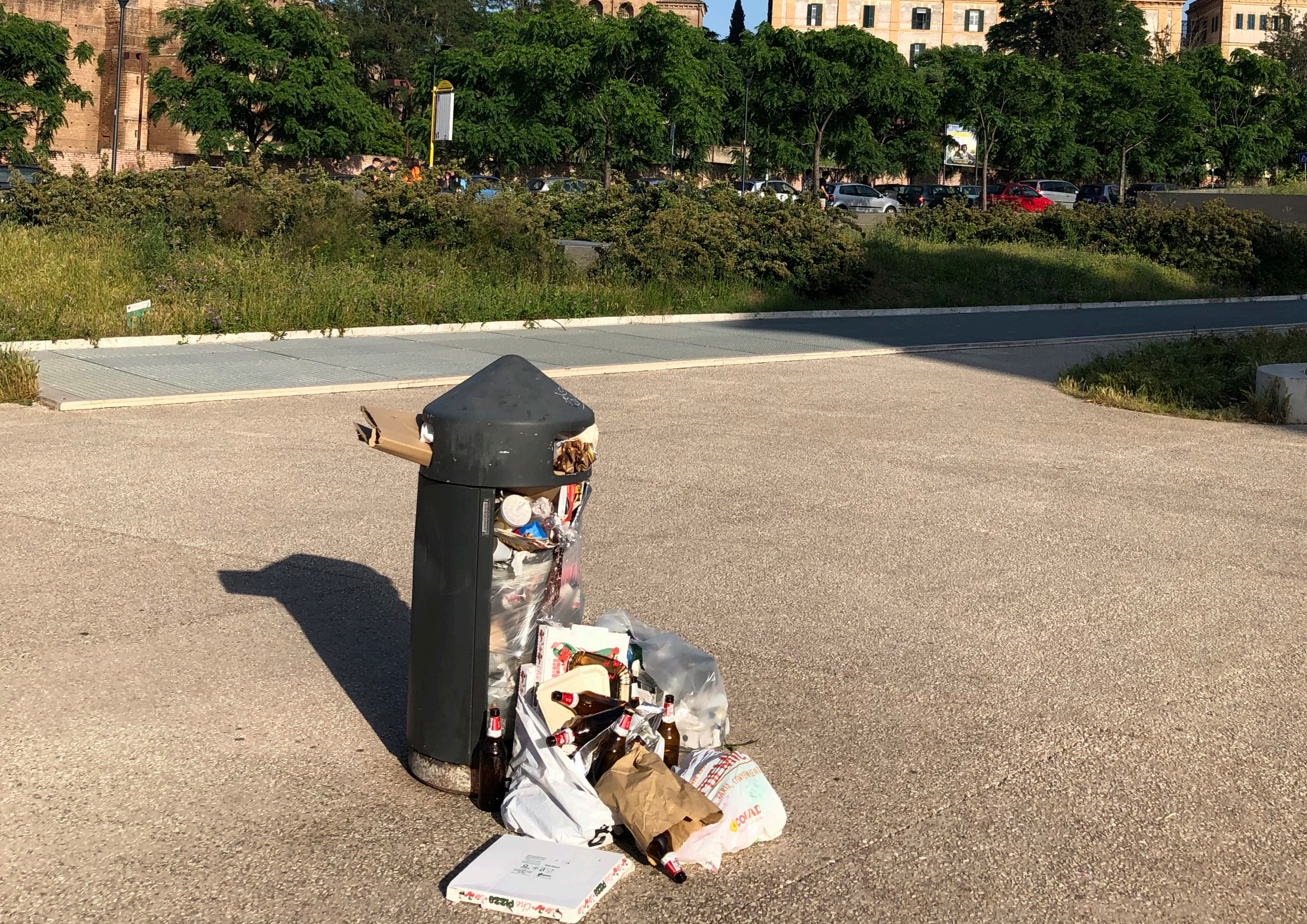
Pizza

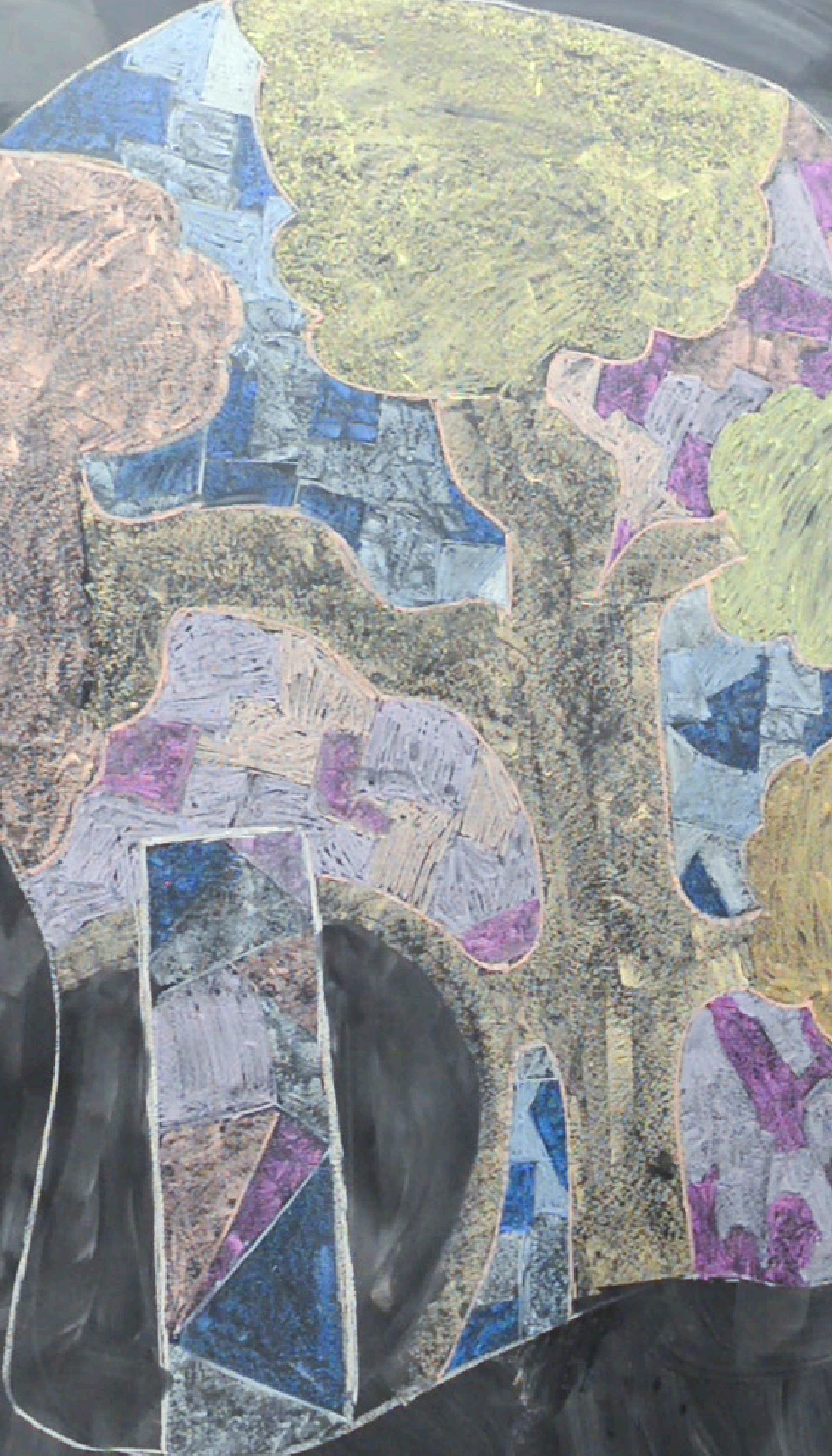

In this context “Dialogues in the square” is developed: an educational project currently involving more than 200 students and about 20 teachers to study and carry out improvements to an important square in the city of Rome.
It involves the schools IC Settembrini (primary and lower secondary) and Liceo Machiavelli based in Rome, in the area of Corso Trieste, Piazza Indipendenza and San Lorenzo.

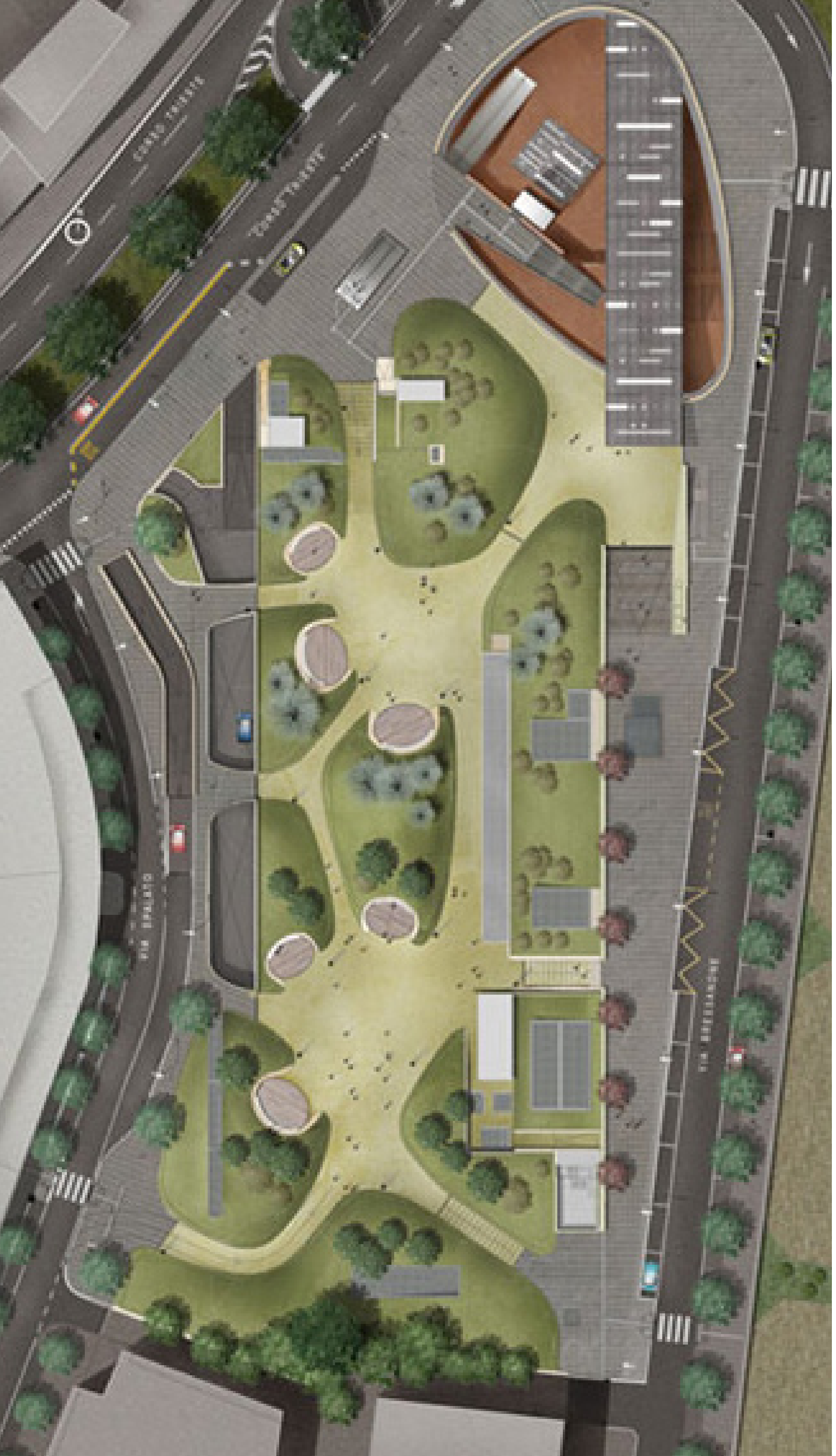

The square is Piazza Annibaliano, an emblematic crossroads of ancient and modern.

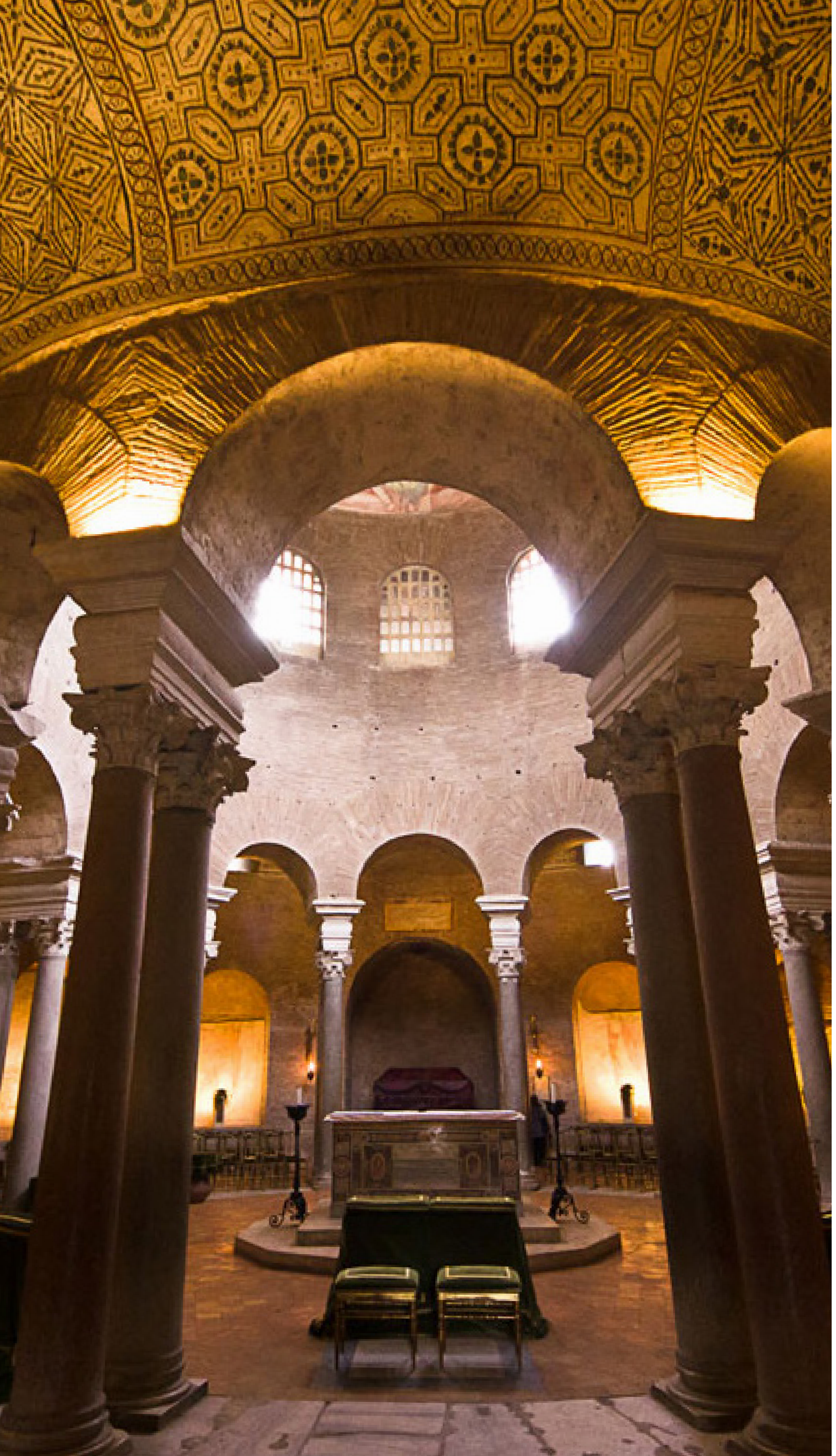

Here the monumental complex of Sant'Agnese Fuori Le Mura (mausoleum of Constance, walls of the Constantinian basilica built in the fourth century, catacombs of Sant'Agnese and the current basilica of Sant'Agnese built in the seventh century) is placed next to the soaring lines of the very busy underground station, opened in 2014 and surrounded by a modern garden, but today already in a state of total degradation.

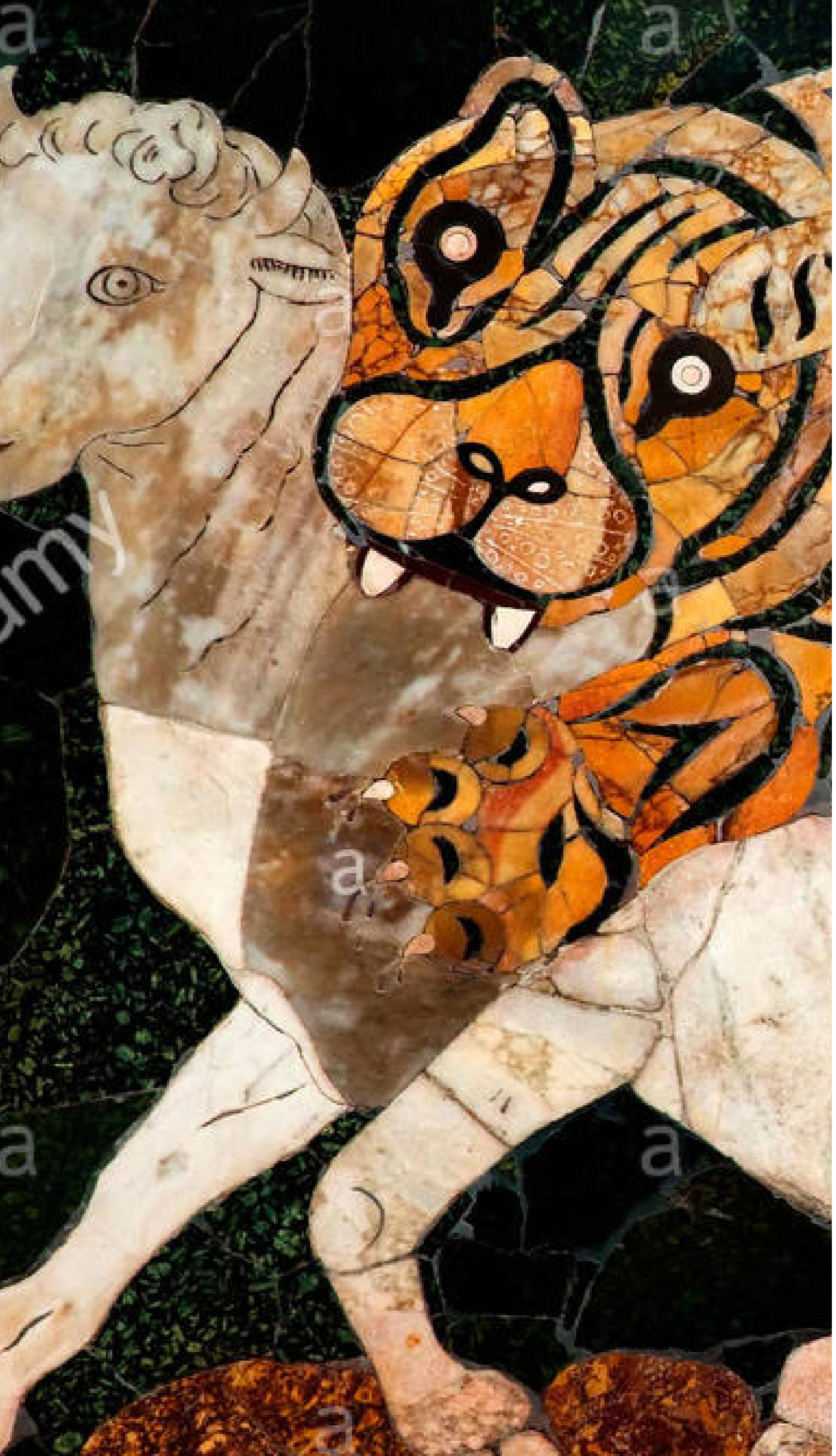

A place of art, stories and meetings, with great artistic and social potential, currently left to itself.

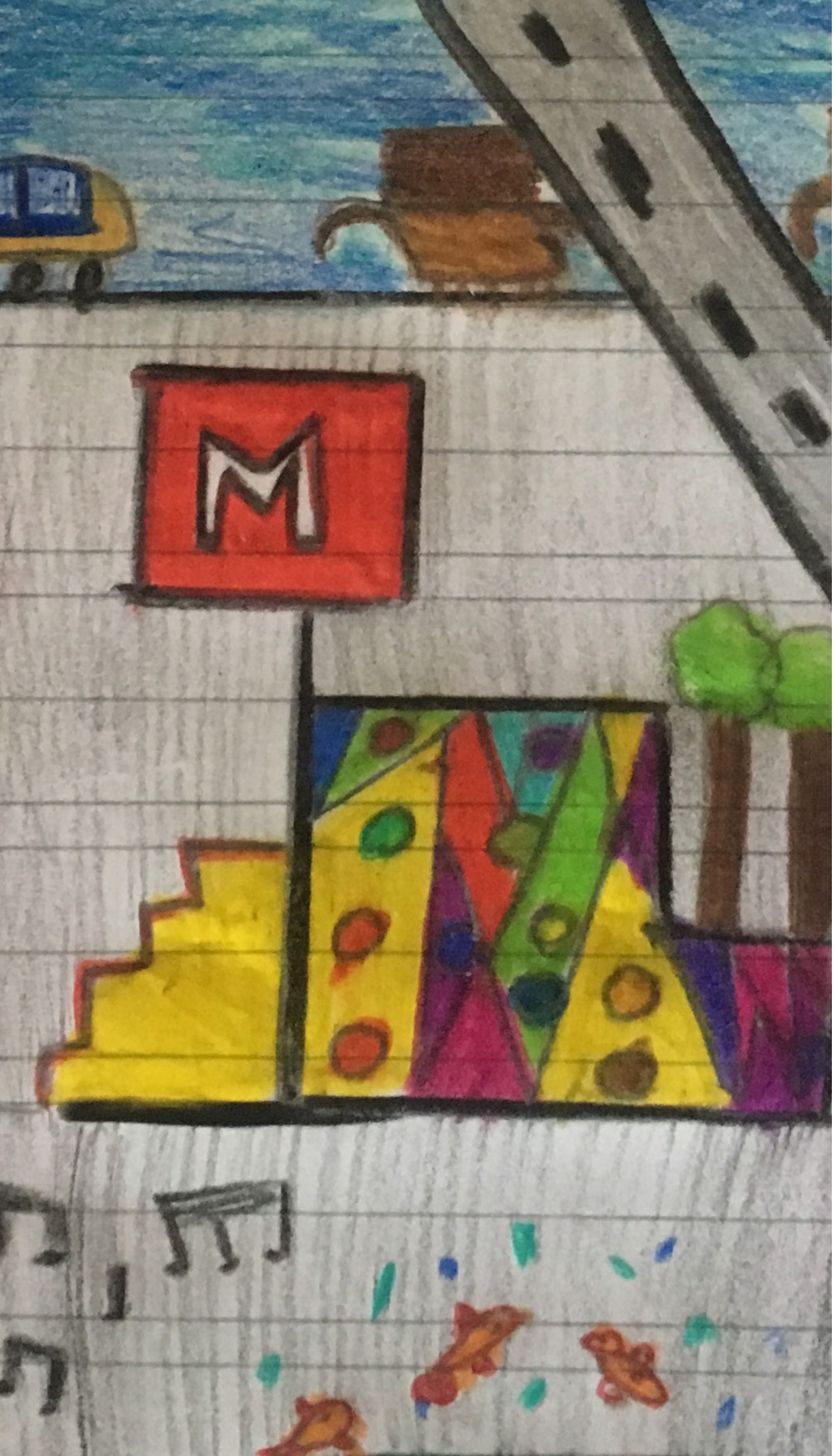

The students began to observe the square, studying it and thinking about ideas on how to improve it. They produced videos and administered surveys. The schools have started a partnership with the II Municipality of Rome, which resulted in a Memorandum of Understanding that contains the blueprint for the work, aligned with the targets of 4 sustainable development goals.

In particular:

- **4 Ensure inclusive and equitable quality education and promote life-long learning opportunities for all (4.7);**
- **11 Make cities and human settlements inclusive, safe, resilient and sustainable (11.4, 11.7);**
- **12 Ensure sustainable consumption and production patterns (12.8, 12.b);**
- **16 Promote peaceful and inclusive societies for sustainable development, provide access to justice for all and build effective, accountable and inclusive institutions at all levels (16.6, 16.7).**

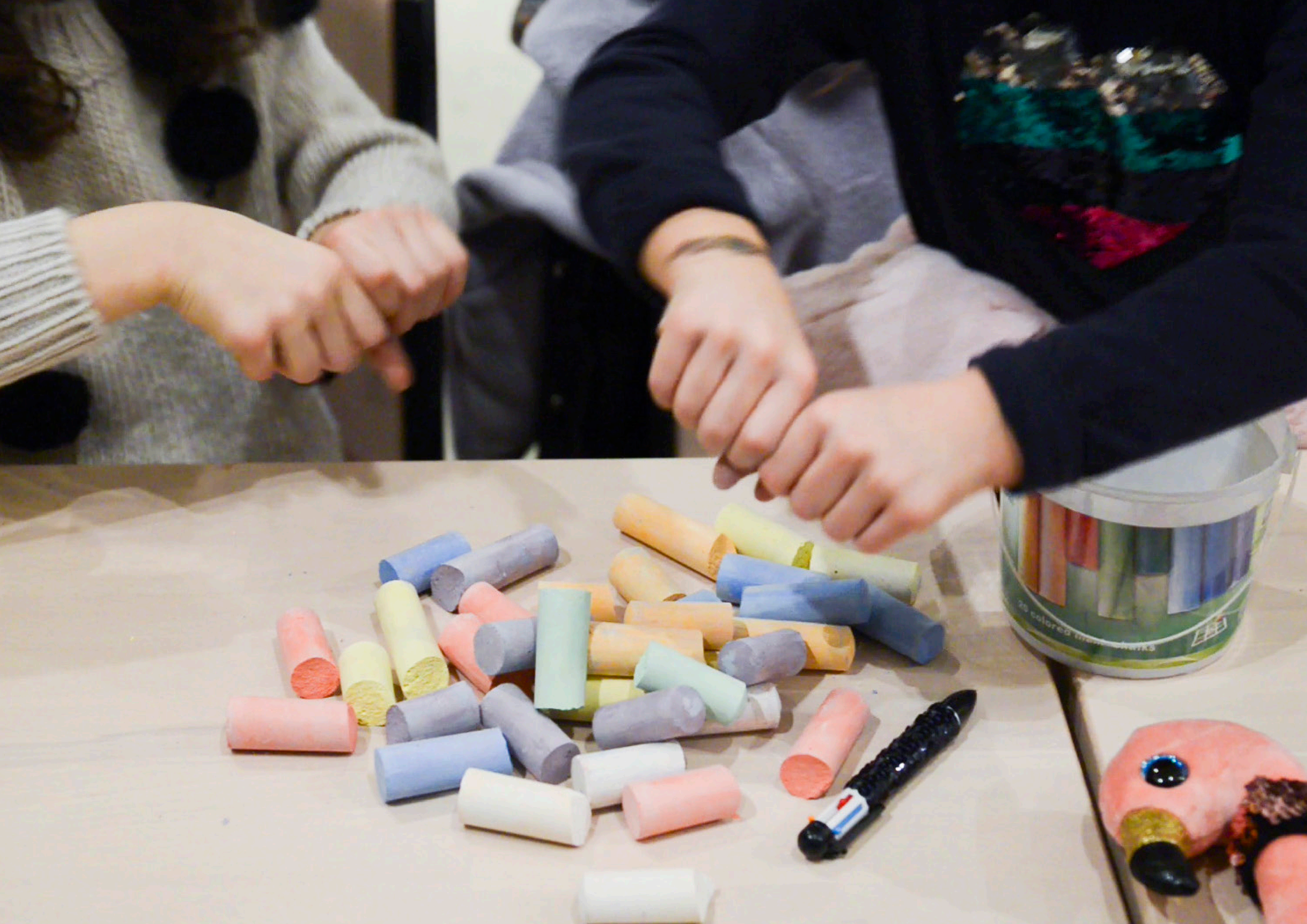

There are four design and intervention areas on the square:

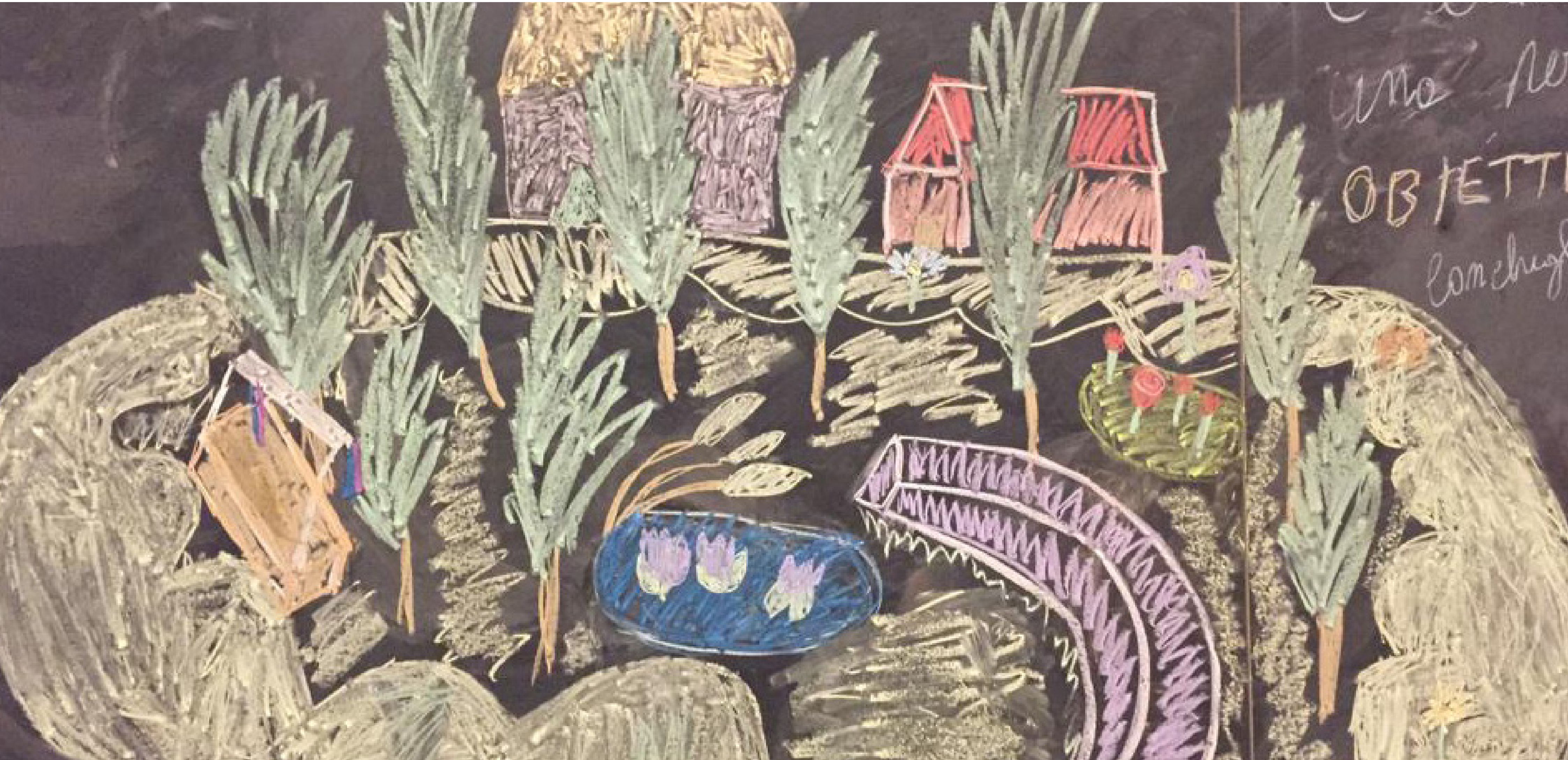

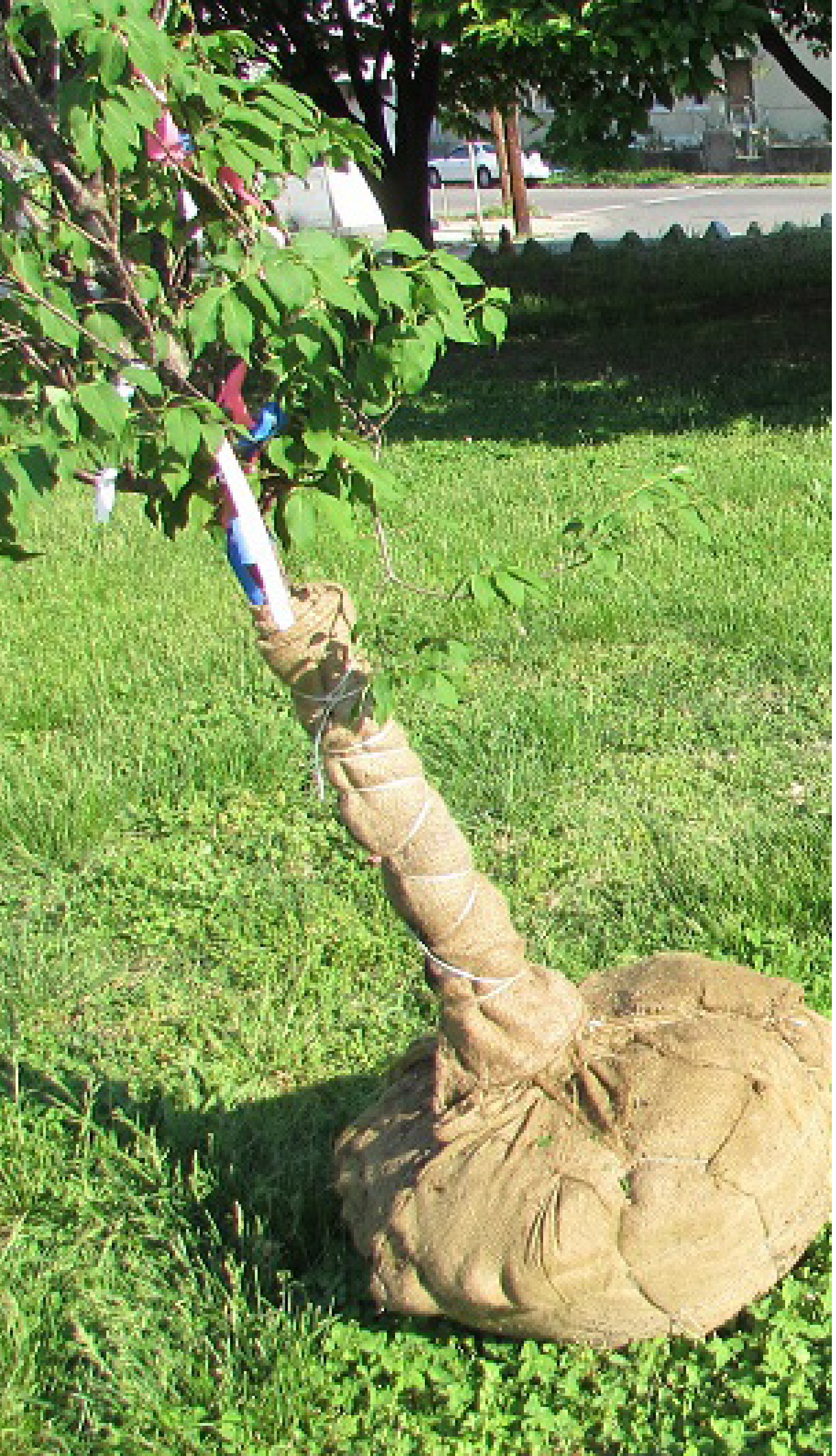

• improve the decorum of the green areas of the square by creating horticultural or decorative cultivation areas;

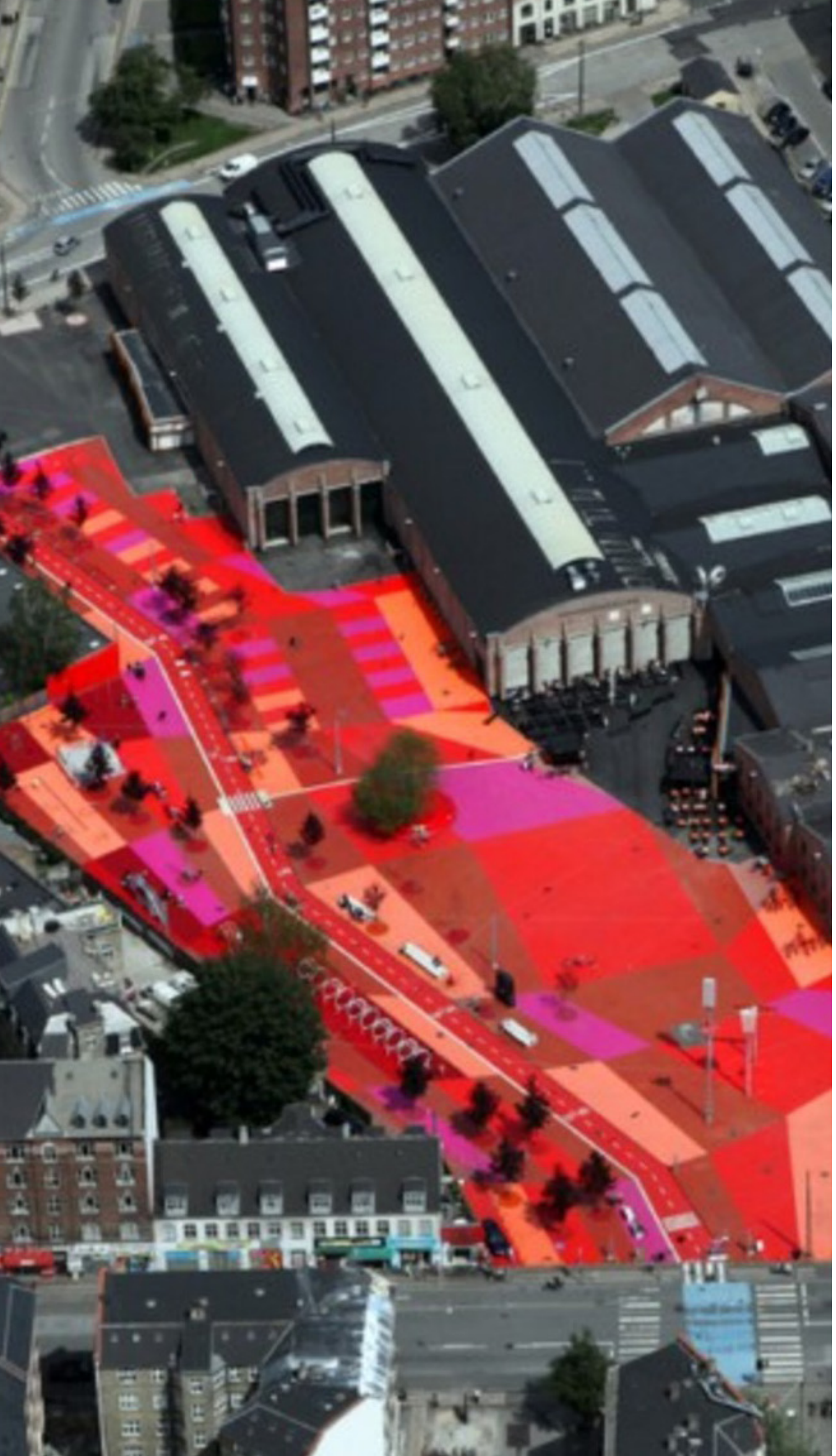

• realize the artistic design of murals in areas to be identified among those currently covered by random graffiti to contrast vandalism and abusive posting and at the same time create works of art in the square;

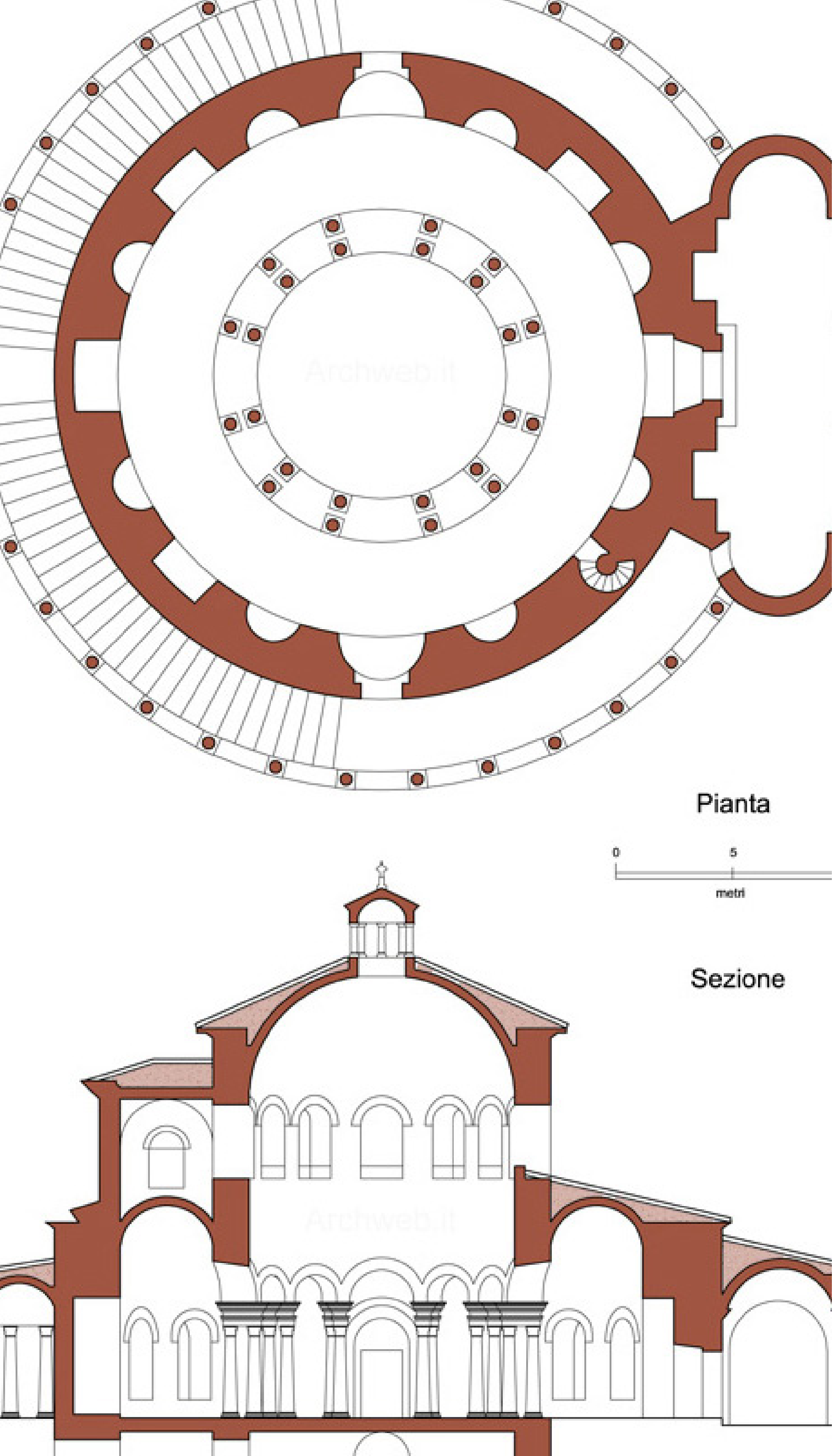

• elaborate reports on the history of the square and its monuments, in order to expose artefacts to make visitors aware of them;

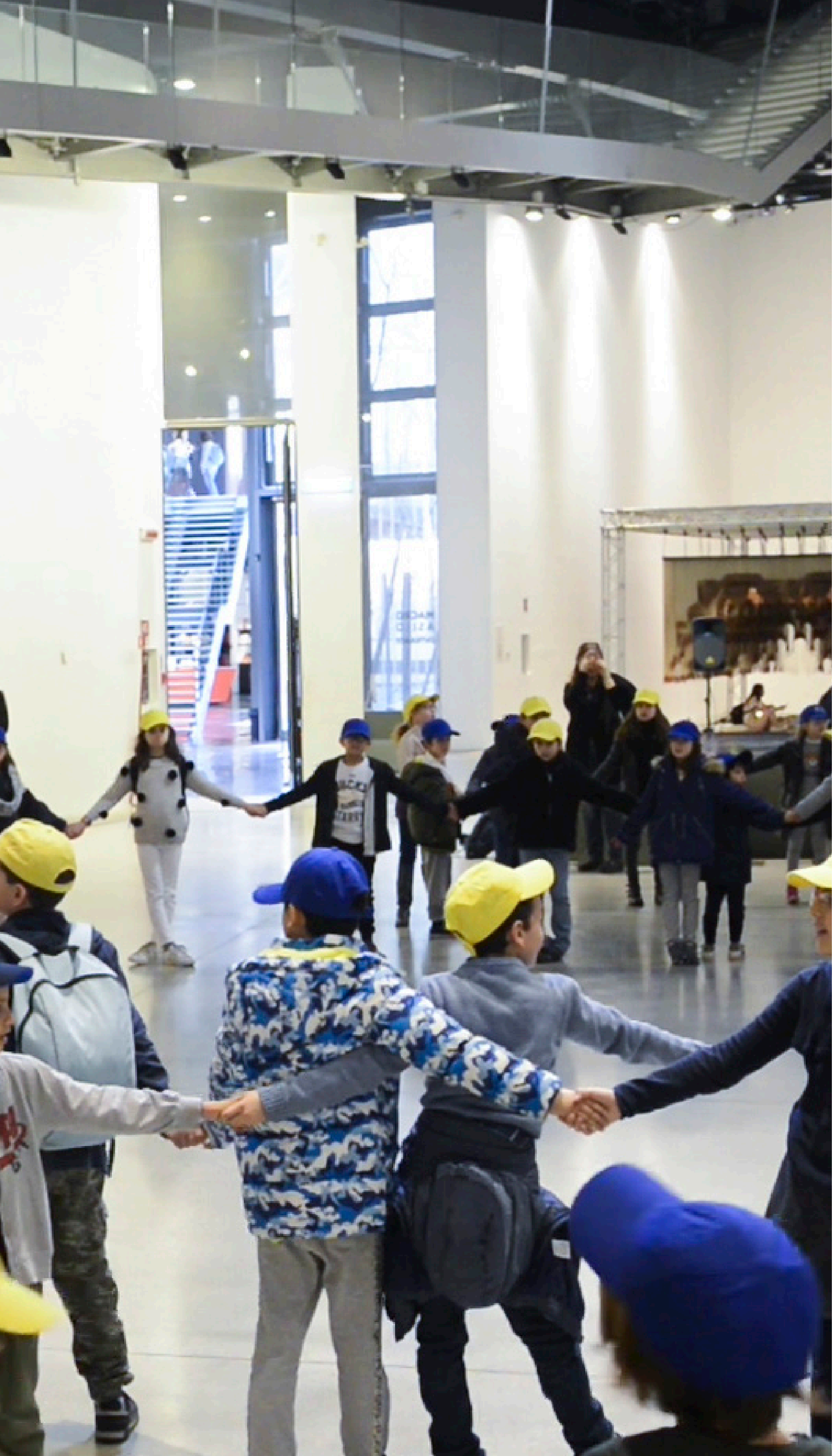

- promote the effective use of the square as an urban space of socialization and sharing, favouring socialization through the realization of events such as Parties, Flash Mob, Art Installations etc.

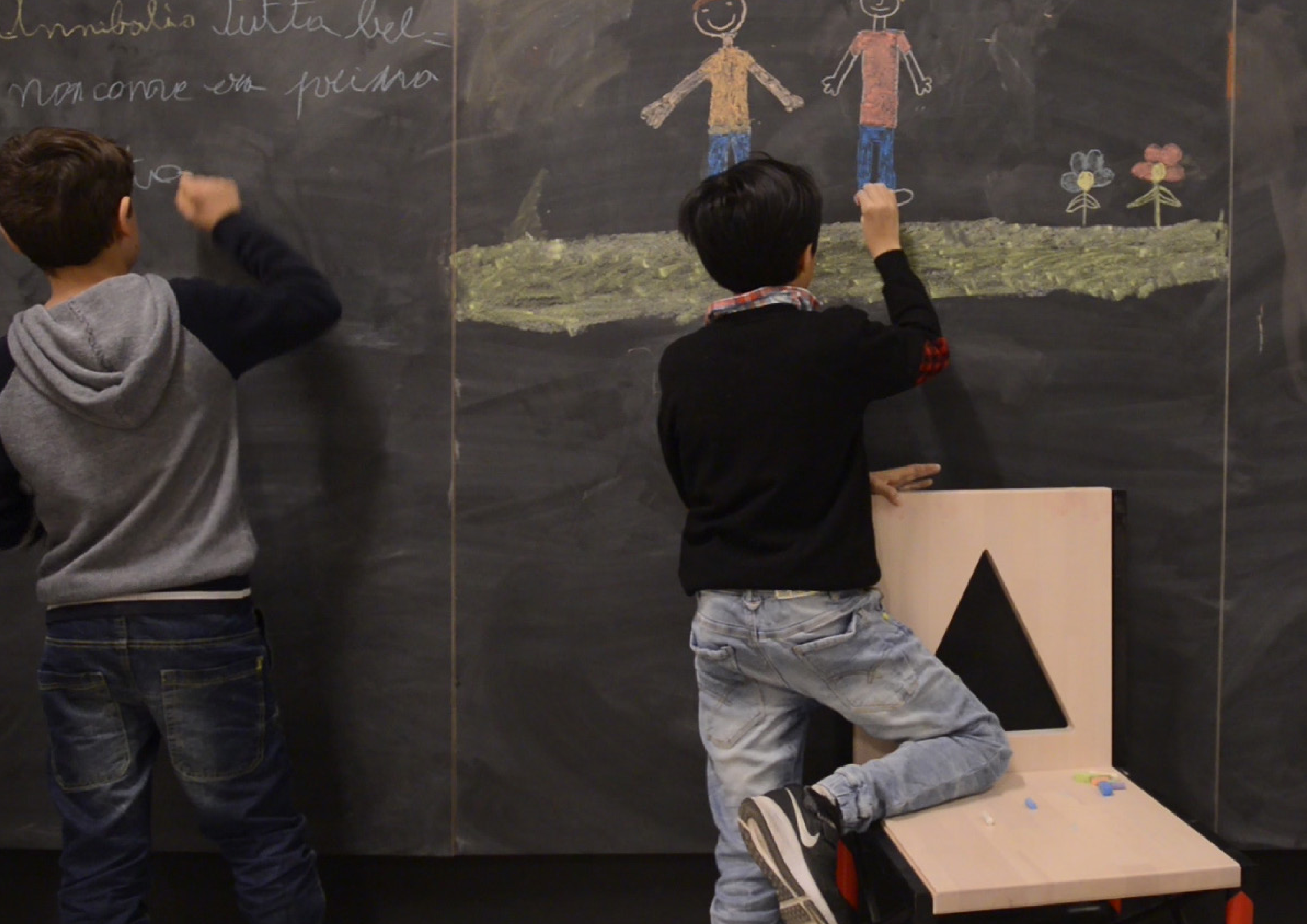

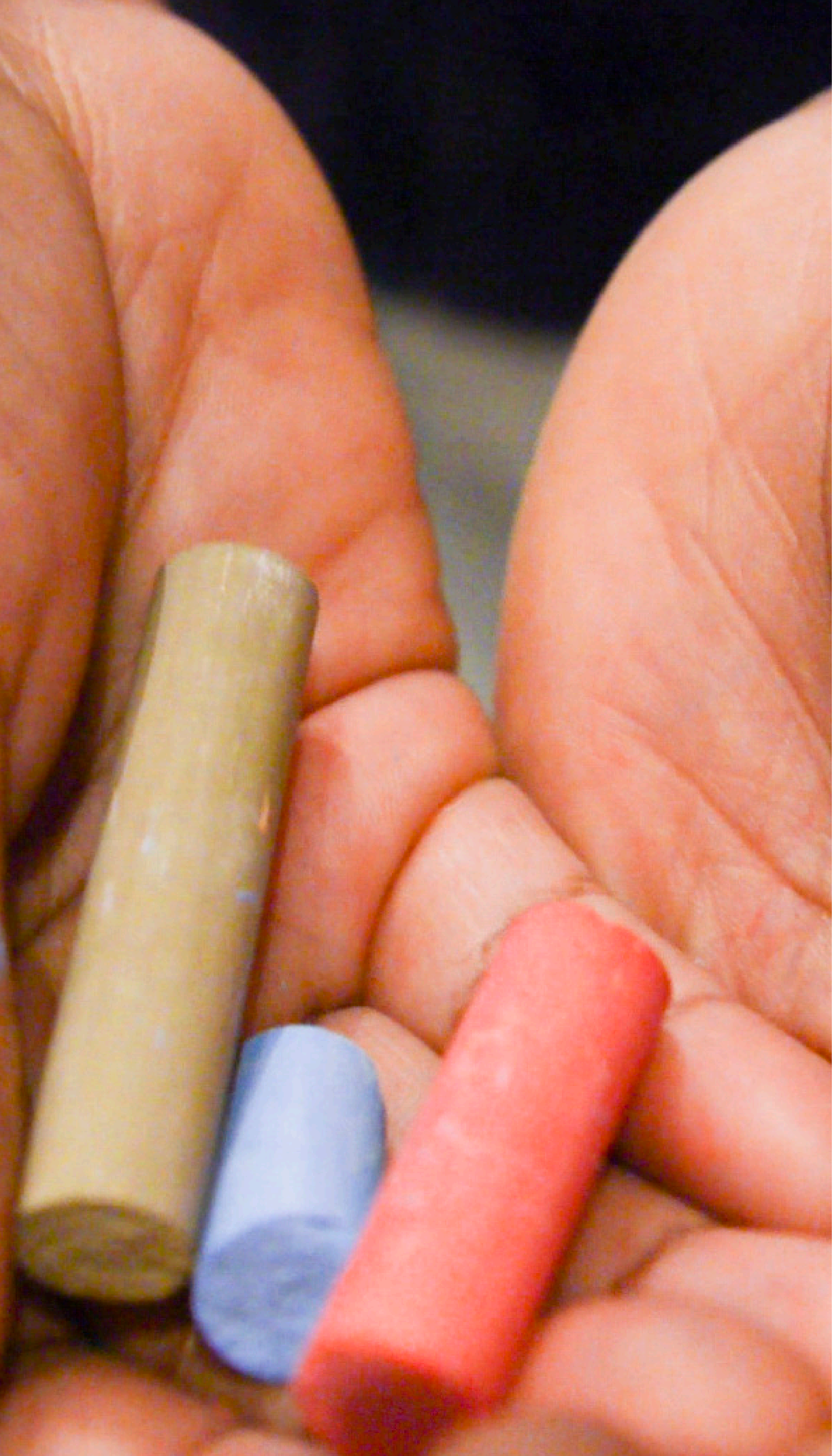

5. SYNERGIES AND INSTRUMENTS

The project grows and the opportunities of the territory offer new synergies, which develop with a snowball effect, offering tools and leading to shared actions.

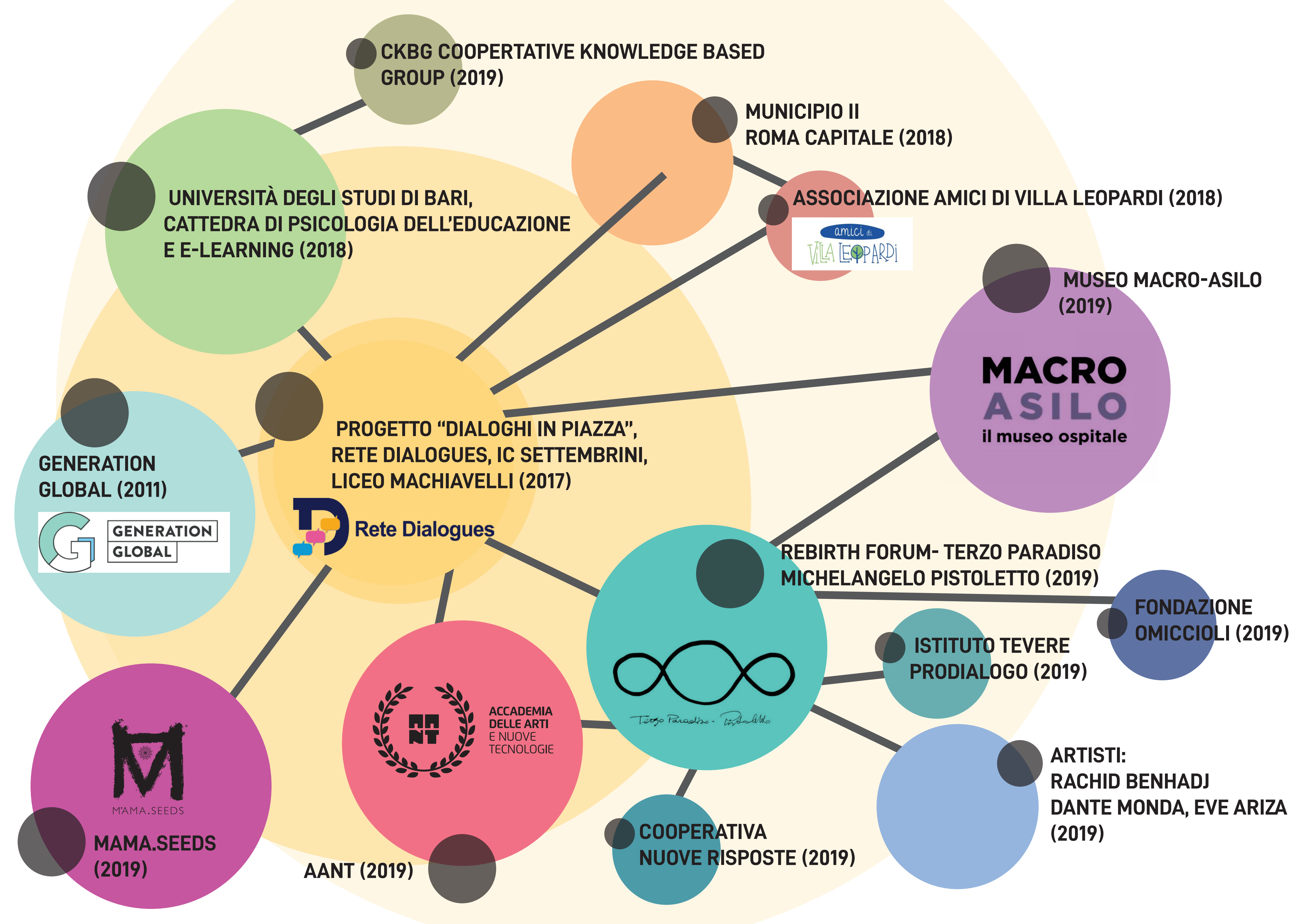
CKBG COOPERTATIVE KNOWLEDGE BASED GROUP (2019)
MUNICIPIO II ROMA CAPITALE (2018)
UNIVERSITÀ DEGLI STUDI DI BARI, CATTEDRA DI PSICOLOGIA DELL'EDUCAZIONE E E-LEARNING (2018)
ASSOCIAZIONE AMICI DI VILLA LEOPARDI (2018)
amici di VILLA LEOPARDI
MUSEO MACRO-ASILO (2019)
MACRO ASILO il museo ospitale
PROGETTO "DIALOGHI IN PIAZZA", RETE DIALOGUES, IC SETTEMBRINI, LICEO MACHIAVELLI (2017)
Rete Dialogues
GENERATION GLOBAL (2011)
GENERATION GLOBAL
REBIRTH FORUM- TERZO PARADISO MICHELANGELO PISTOLETTO (2019)
FONDAZIONE OMICCIOLI (2019)
ISTITUTO TEVERE PRODIALOGO (2019)
ACCADEMIA DELLE ARTI E NUOVE TECNOLOGIE
AANT (2019)
MAMA.SEEDS
MAMA.SEEDS (2019)
COOPERATIVA NUOVE RISPOSTE (2019)
ARTISTI: RACHID BENHADJ DANTE MONDA, EVE ARIZA (2019)

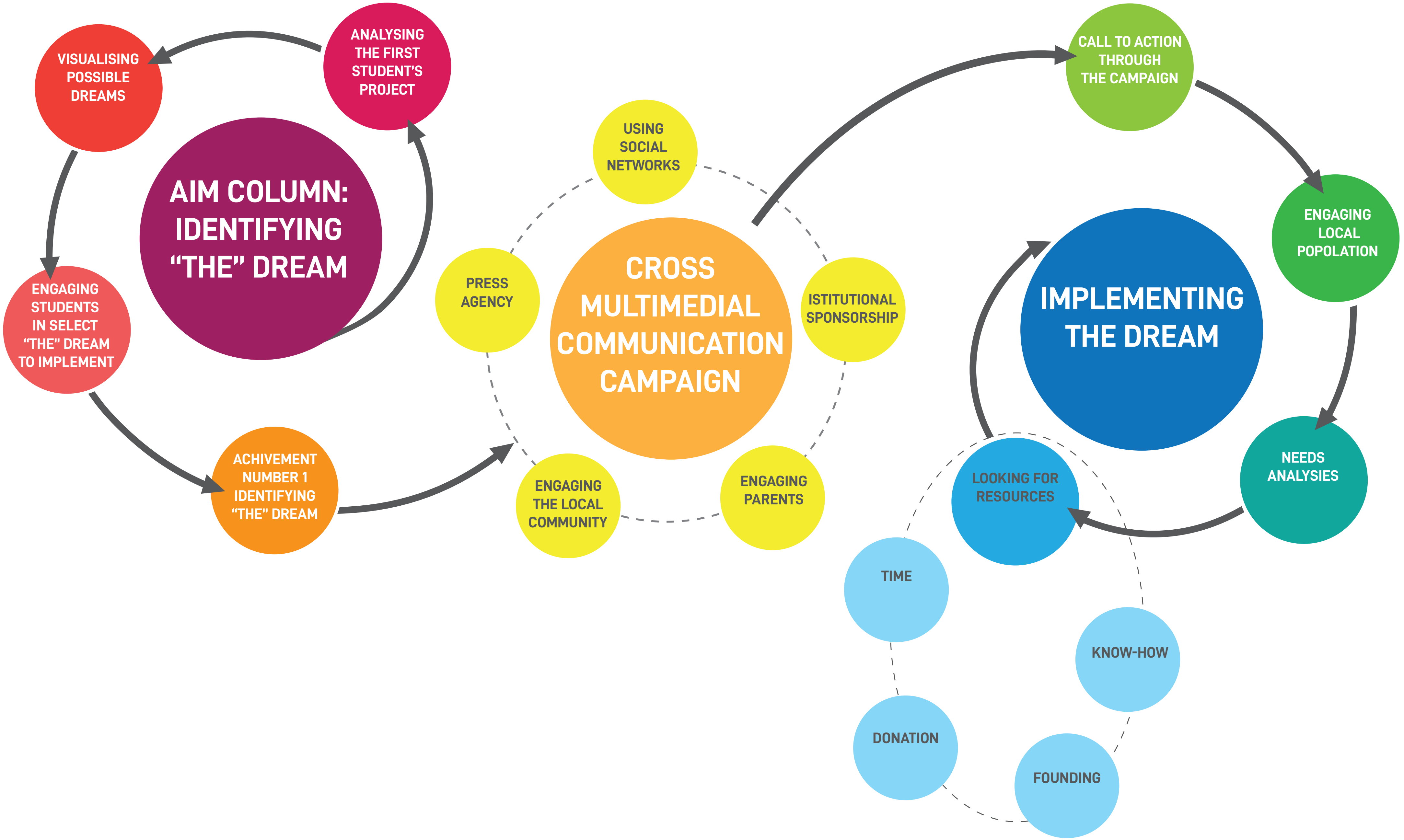
AIM COLUMN: IDENTIFYING "THE" DREAM
ANALYSING THE FIRST STUDENT'S PROJECT
VISUALISING POSSIBLE DREAMS
ENGAGING STUDENTS IN SELECT "THE" DREAM TO IMPLEMENT
ACHIVEMENT NUMBER 1 IDENTIFYING "THE" DREAM
CROSS MULTIMEDIAL COMMUNICATION CAMPAIGN
USING SOCIAL NETWORKS
PRESS AGENCY
ISTITUTIONAL SPONSORSHIP
ENGAGING THE LOCAL COMMUNITY
ENGAGING PARENTS
IMPLEMENTING THE DREAM
CALL TO ACTION THROUGH THE CAMPAIGN
ENGAGING LOCAL POPOLATION
NEEDS ANALYSIES
LOOKING FOR RESOURCES
TIME
KNOW-HOW
DONATION
FOUNDING

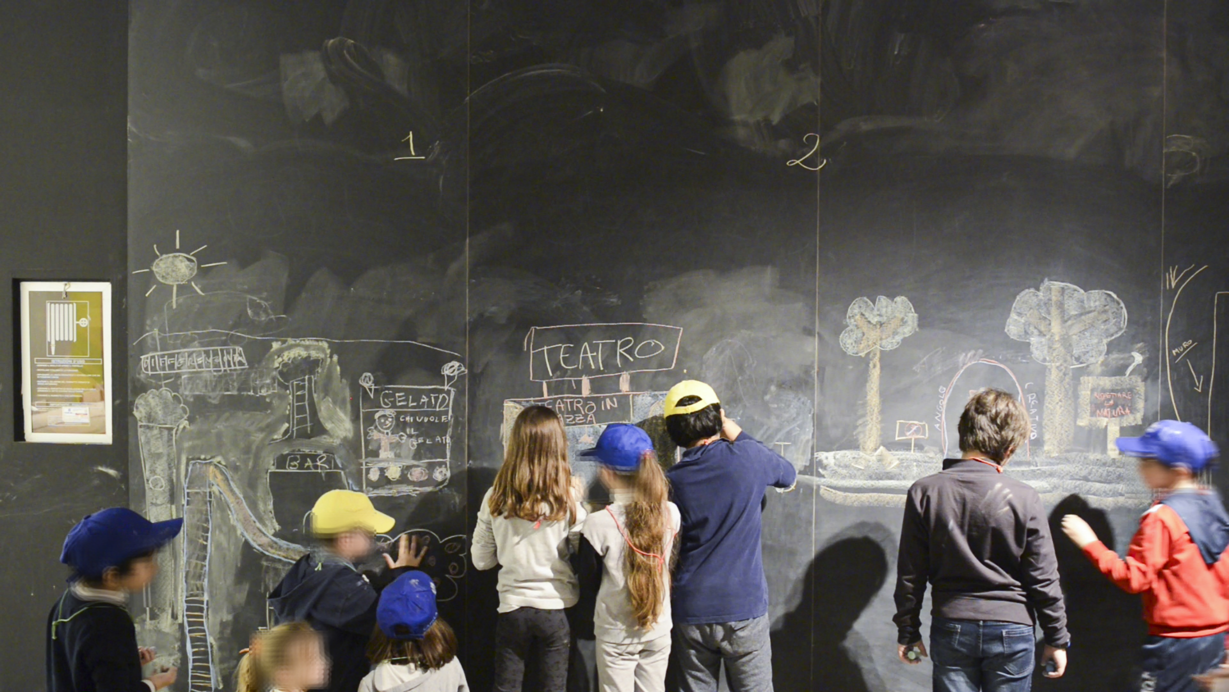
1
2
TEATRO
GELATO
BAR
MURO

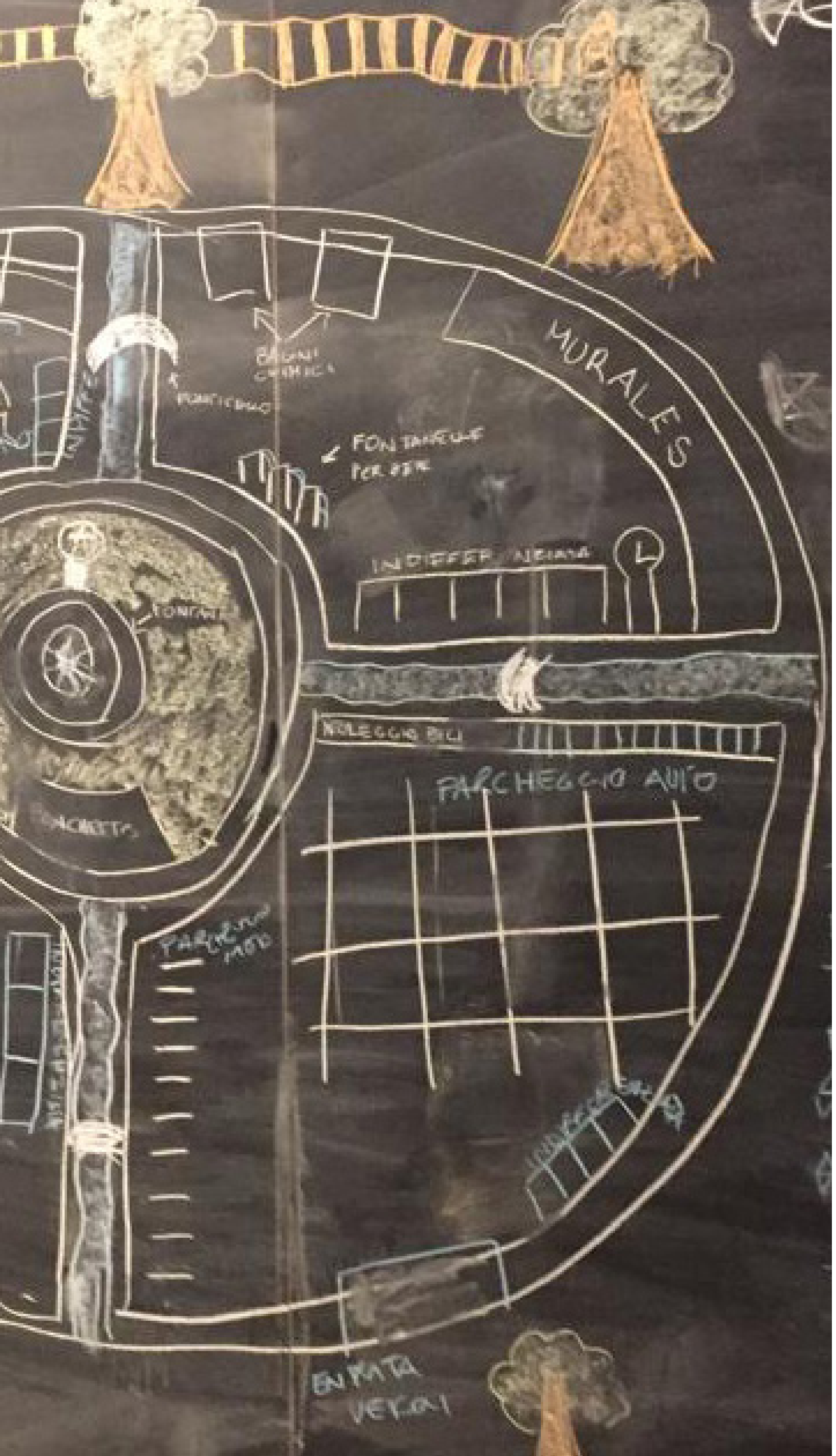

6. TO THE BLACKBOARD!

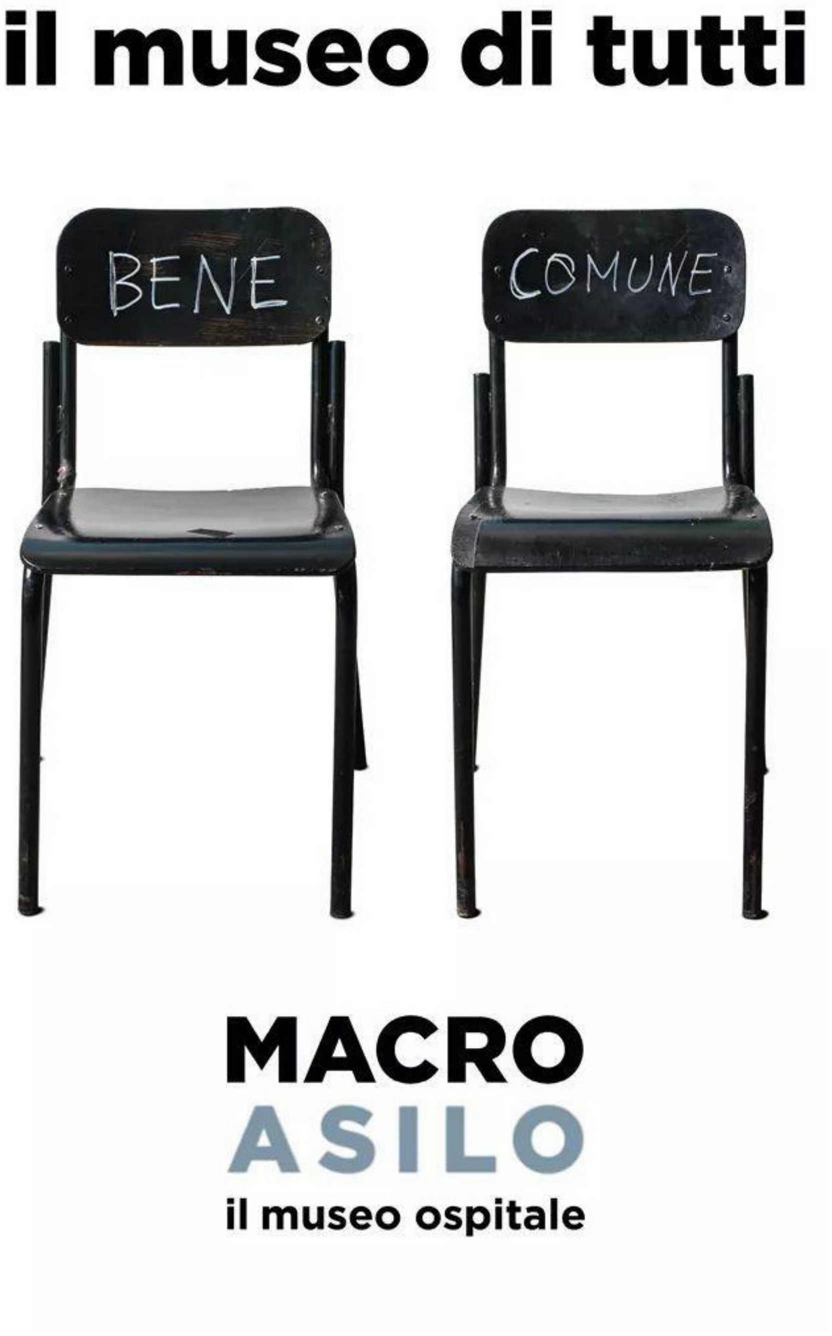

The Dialogues in the Square project is in fieri and develops according to the opportunities that emerge from the network of actors involved.
Here we focus on a particularly significant initiative that is taking place at the MACRO-ASILO Museum, in synergy with the Rebirth Forum-Third Paradise.

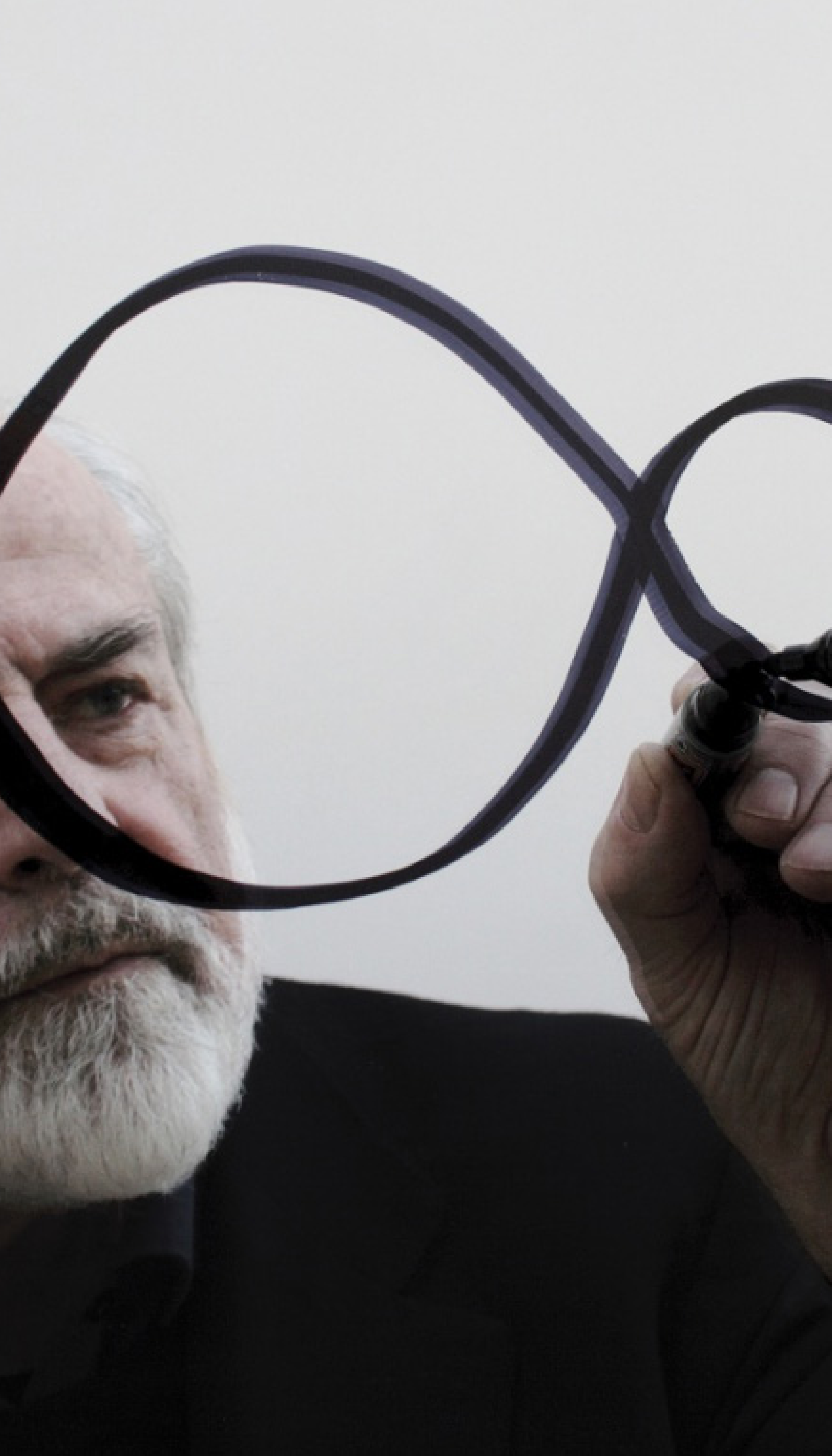

The Third Paradise is the great myth that leads everyone to assume a personal responsibility in the global vision. The word paradise comes from the ancient Persian language and means “protected garden”. We are the gardeners who must protect this planet and take care of the human society that inhabits it. (Michelangelo Pistoletto, 2003)

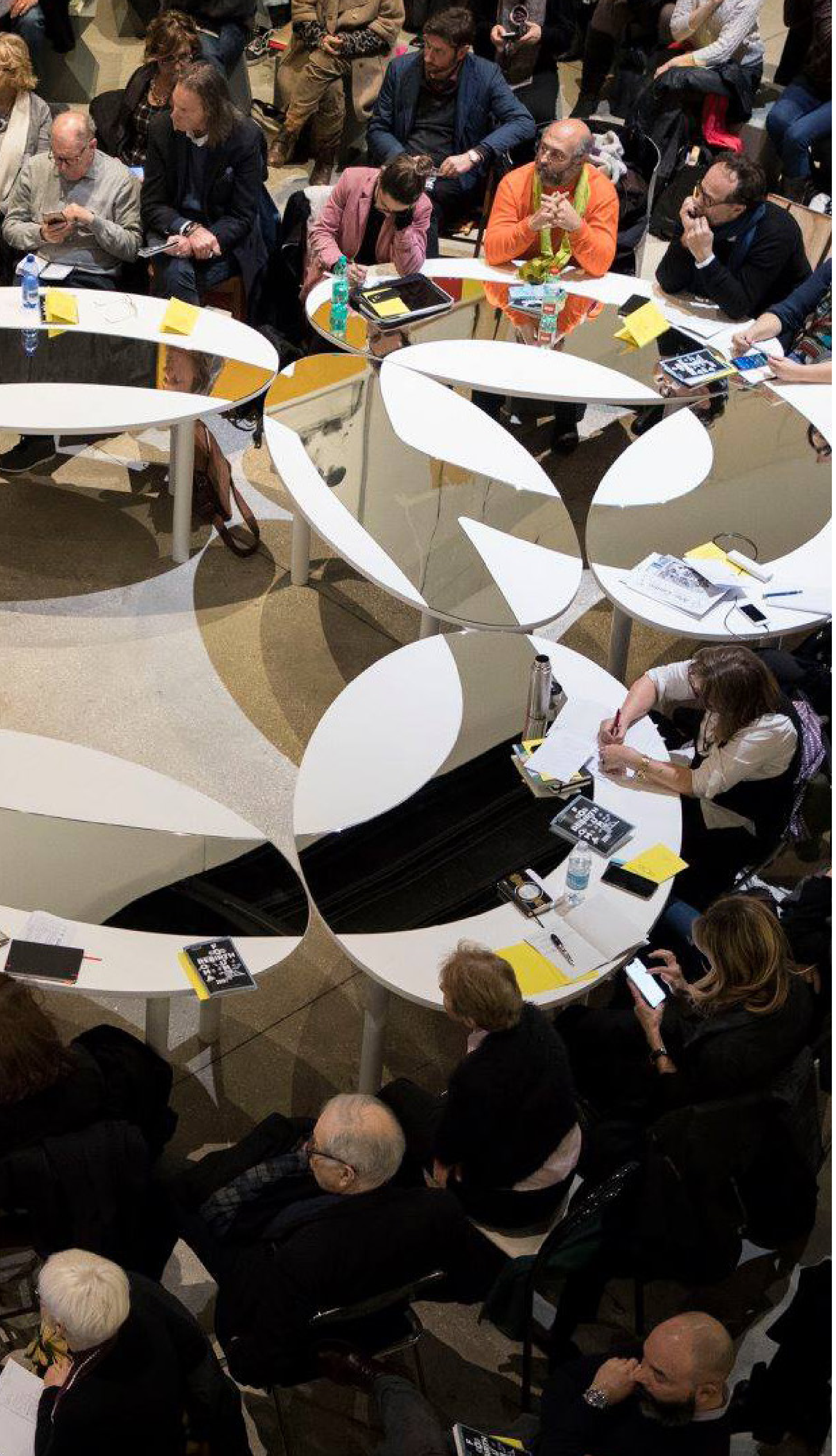

The Rebirth Forum in Rome
is an event that aims to unite the forces between public and private organizations and institutions to experiment everyday sustainability practices in the different areas of human society: from production to intercultural dialogue, from design to agriculture, from culture to health, etc.

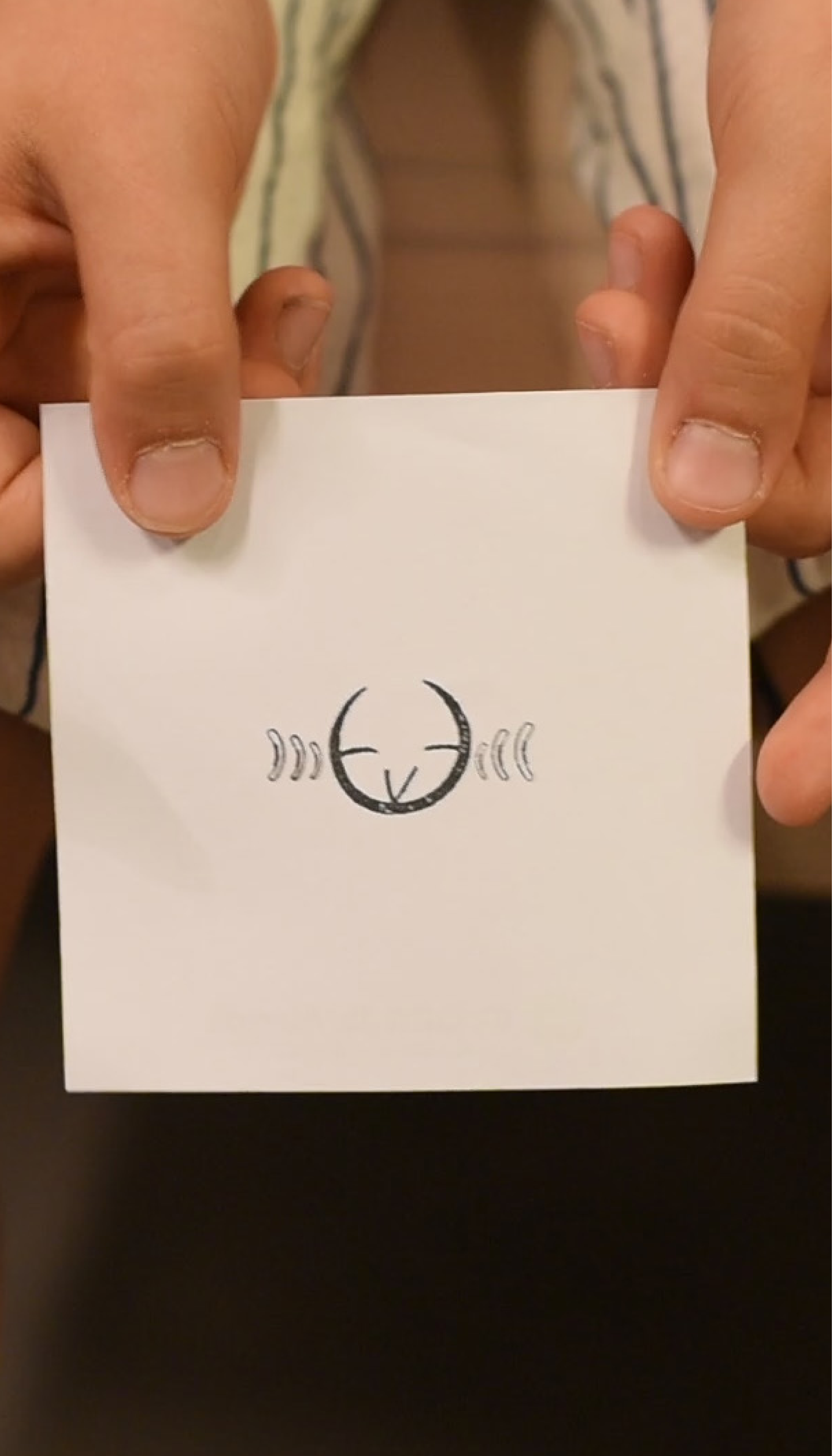

In collaboration with the MACRO Asilo, it brings together one hundred representatives of many organizations active in the territory, around "the tables of the Third Paradise".

The Macro Asilo project offers a valuable work tool:
the "words room", a creative work space equipped with a 22x4 meter blackboard, where it is possible to organise workshops

Each of the 8 classes participates in one or two workshops that are located within the Macro Asilo DICTIONARY paths, which aim to decline some key terms of contemporary life.

In the 9 meetings the dictionary word is approached in different ways and the students engage with an artist or an expert. Students prepare beforehand at school for the artist / expert they are going to meet to address the theme of the "square", they elaborate questions, proposals to discuss. The expert in turn prepares an interactive activity that involves the use of the blackboard.

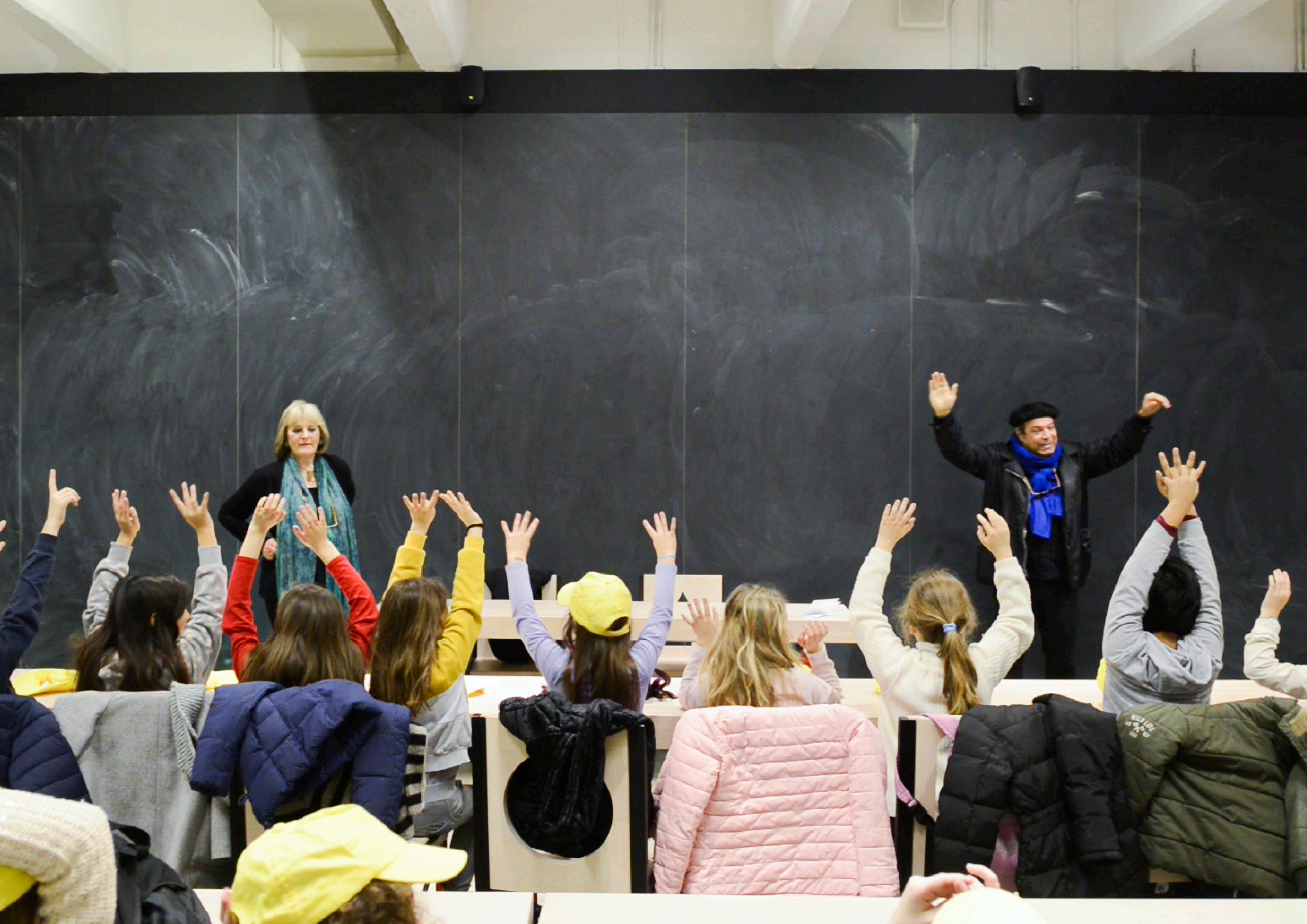

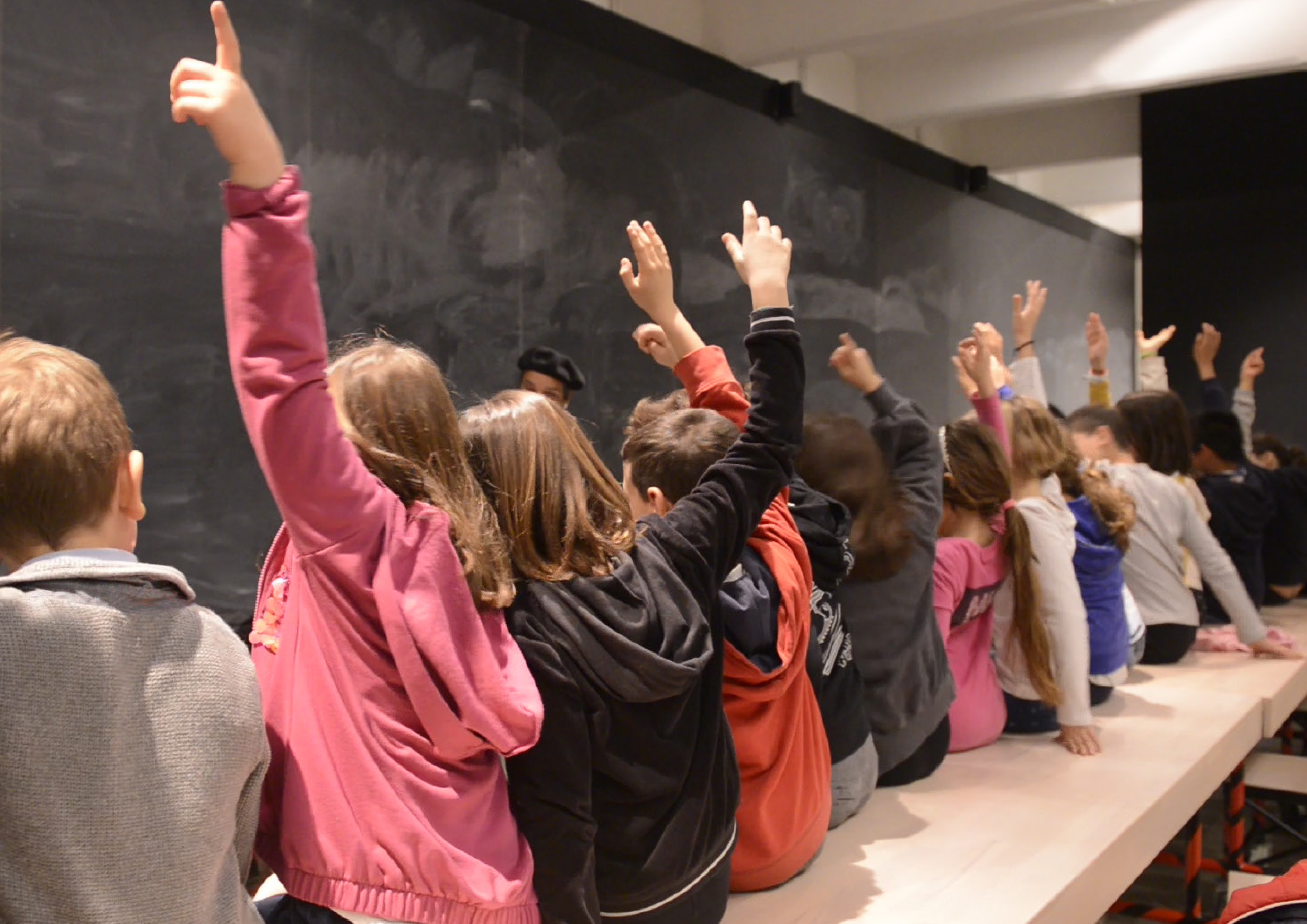

These are some "themes" developed, each with a different group of students, sometimes combining classes of different ages:

# square 1 stories in the square (with director Rachid Benhadj).
Delivery: draw stories you wish you could live in Piazza Annibaliano

#square 2 red threads in the square (with atelierista Enza Policardi)
Delivery: which new objects in the squadre an how they link to create a good solutions to improve the environment

#square 3 words and sounds in the square (with blogger and musician Dante Monda)
Delivery: create a rap song to play in a performance in the square

#square 6 square 4.0 (with designers Giancarlo Cutello and Ilaria Narducci)
Delivery: examine the current project of the square and assess it in the light of other international experiences

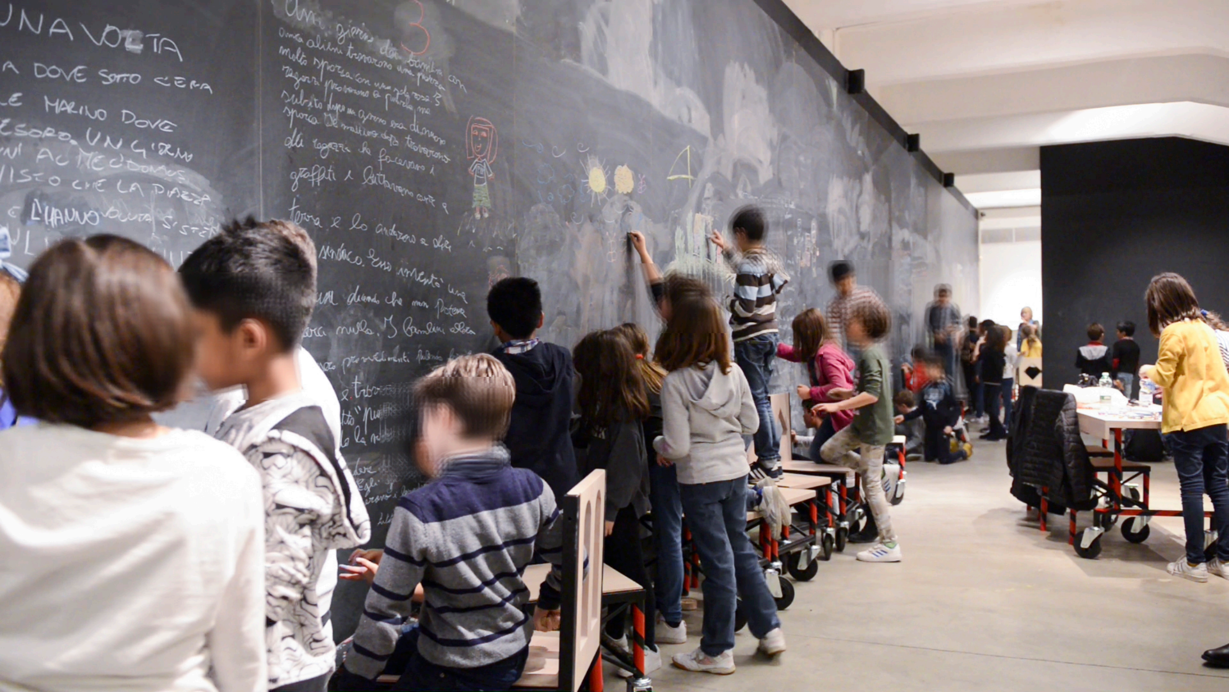

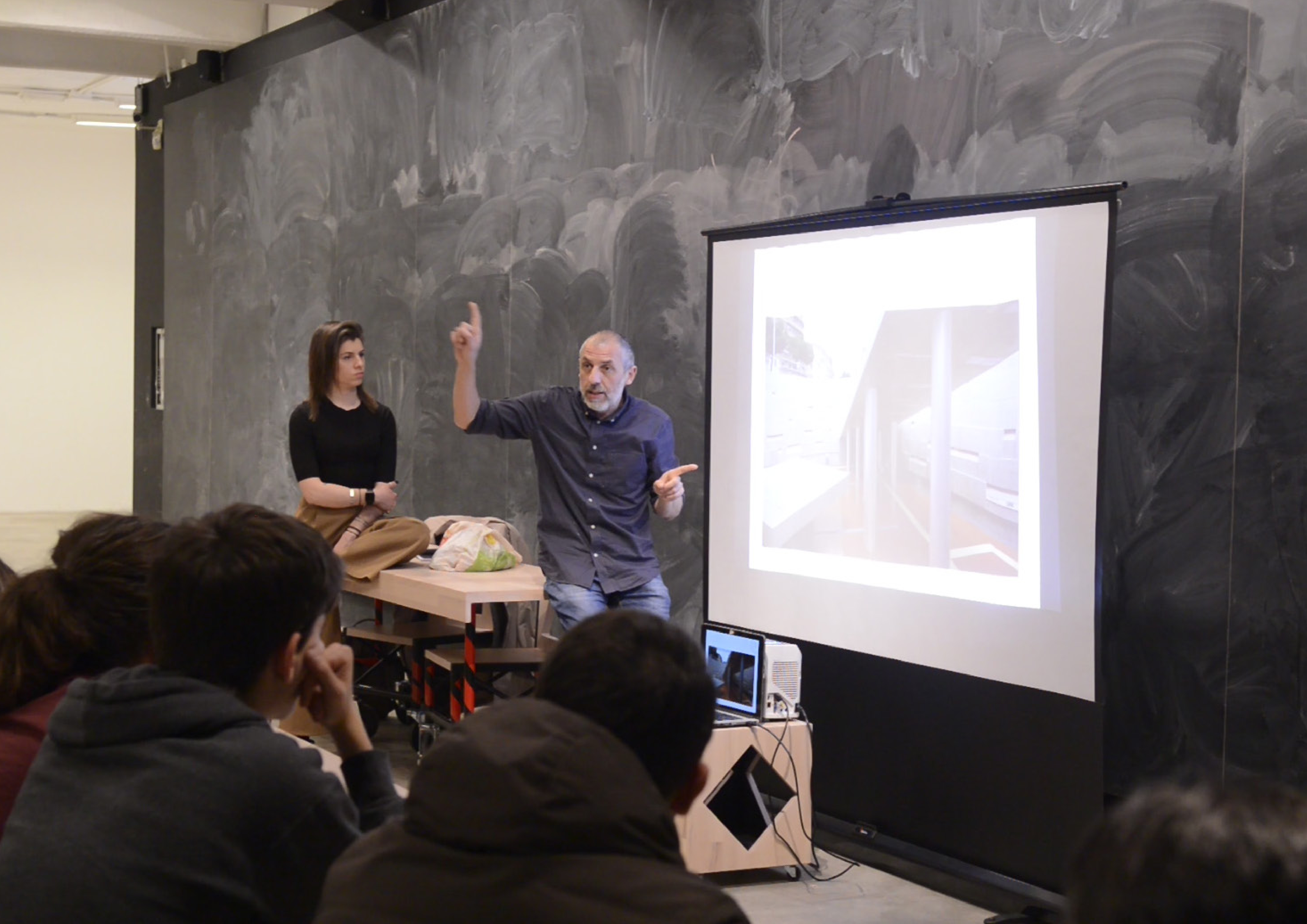

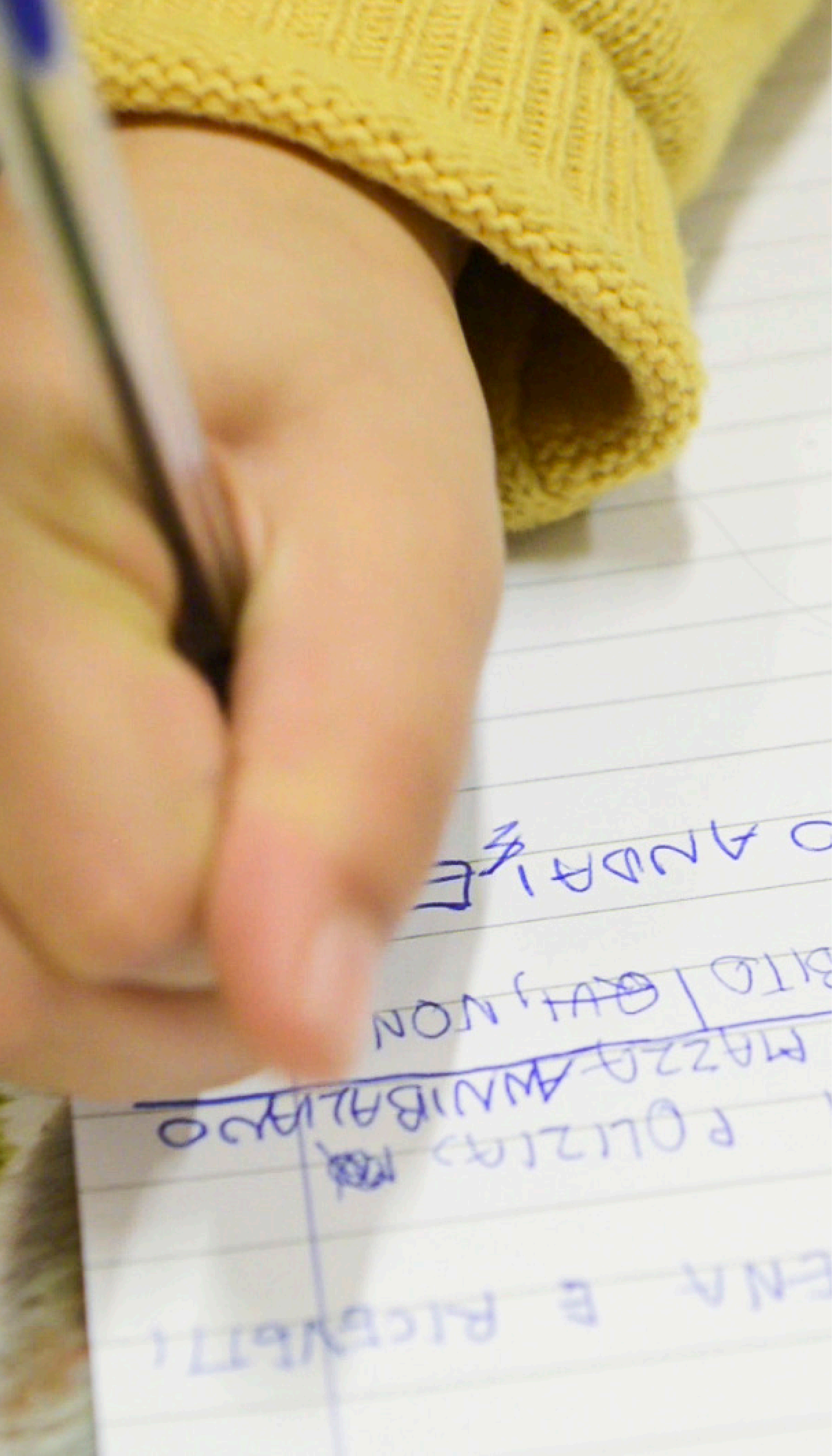

7. CRITICAL ANALYSIS OF THE PROCESS AND CRITICAL INCIDENTS

Some reflections

ENVIRONMENT, SPACE AND TOOLS

seem to have a great impact on the creative processes and on the performance of the students, but also on the attitude of the teachers. The blackboard-room space is explored in all its potential: to position oneself in a different way and draw, discuss, observe, making one's emotions more alive.

It is the space with objects in it that becomes the leader of the action, facilitating processes of self-regulation.

This allows us to understand how interesting and important it is for the educational environment to include learning moments in spaces that are different to the regular classroom context, where the "genius loci" can be different: a drawing in the square sitting on benches, a story written in the park sitting on the grass, a reading made under a tree or peeling potatoes in silence to reflect (Eve Ariza 2019).

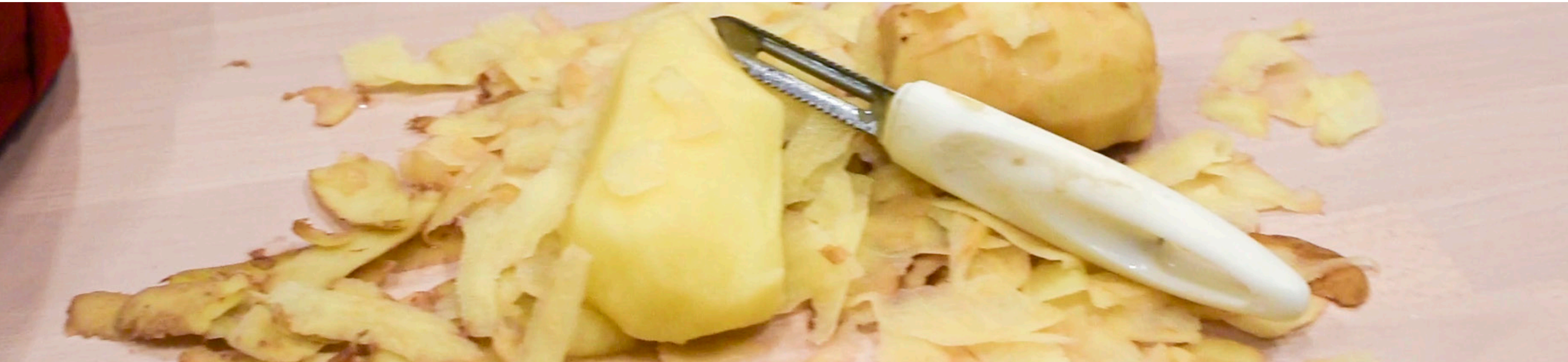

WORKING WITH ARTISTS AND EXPERTS

preparing to meet an expert who has been invited for their work, for their particular ability in a certain sector or for a particular need for living knowledge, which is felt within a project, is an art to be cultivated with care.

In this case the opportunity was created for the students to be prepared and to have the taste of “feeling up to the situation” and to have the curiosity to ask appropriate questions.

It is not a case of passive listening and fruition of the story, but rather a concerted action that produces an intense interaction between students and experts/artists.

Here the artist or expert is invited to participate in a path, to recommend solutions, strategies.

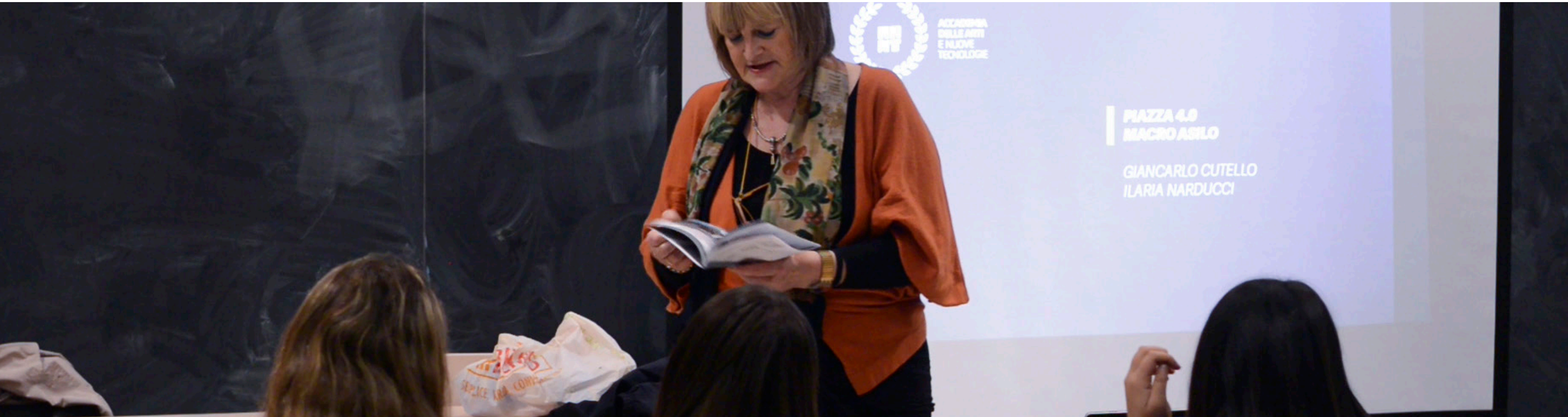

GROUP DYNAMICS

The context conceived with the combination of: artist / expert, particular place (the "words" room) and prepared students (the interdisciplinary work in preparation), actually has influence on cognitive processes but also on group dynamics, creating a sort of virtuous circle.

Despite the frenzied and at times noisy climate, a remarkable capacity for self-regulation is realized where people discuss and collaborate.
The blackboard with its gigantic dimensions becomes an important leader, who seems to "creatively" tease regular stereotypes by overturning traditional roles and relationships and giving priority to the interest of the task.

The monitoring in progress reveals that the students, explaining their satisfaction in regards with the project, cite as a predominant reason the outlook of a shared work stemming from different points of view.

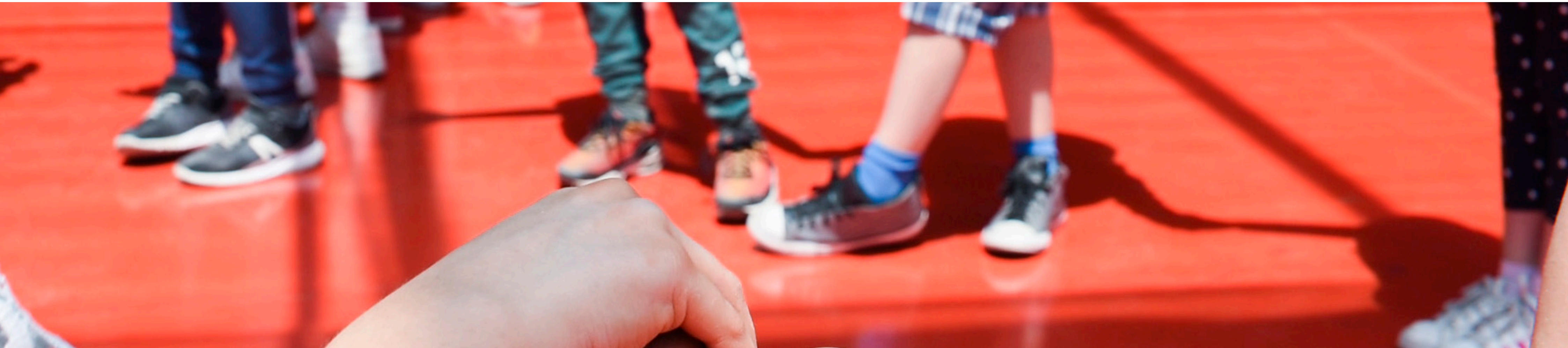

THE DOCUMENTATION

The interest shown for the task, then tested in its execution, also translates into a desire to document in order to "show", reproduce, recount with documents in hand.

The blackboard is a contradictory space: large and solid in its materiality and ability to welcome the work of many, it becomes fragile in its ability to preserve information.

The work thus becomes an opportunity to think about "how to tell" an experience, how it is communicated to others, what technological tools and expertise are needed to be able to adequately represent the details.

From the use of the most classic and traditional instrument, an authentic technological need develops.

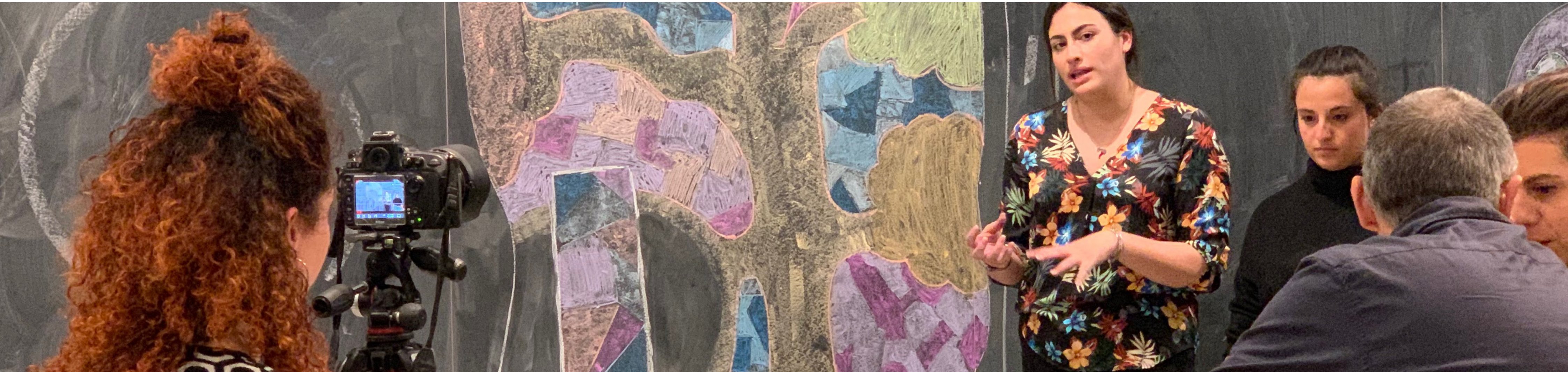

CRITICAL INCIDENTS

The experience “at the blackboard” proves to be rich of “critical incidents”, those unexpected events that, observed with an expert pedagogical eye, allow us to understand in a new and deeper way what happens in the learning process (Tripp, 1993- 2012).

In each meeting there were sudden and often enlightening outings by the most closed and taciturn students and the performances of the weakest sometimes surprised the teachers and the other students.

A theme to be explored that made the rich possibilities of observation of the context evident.

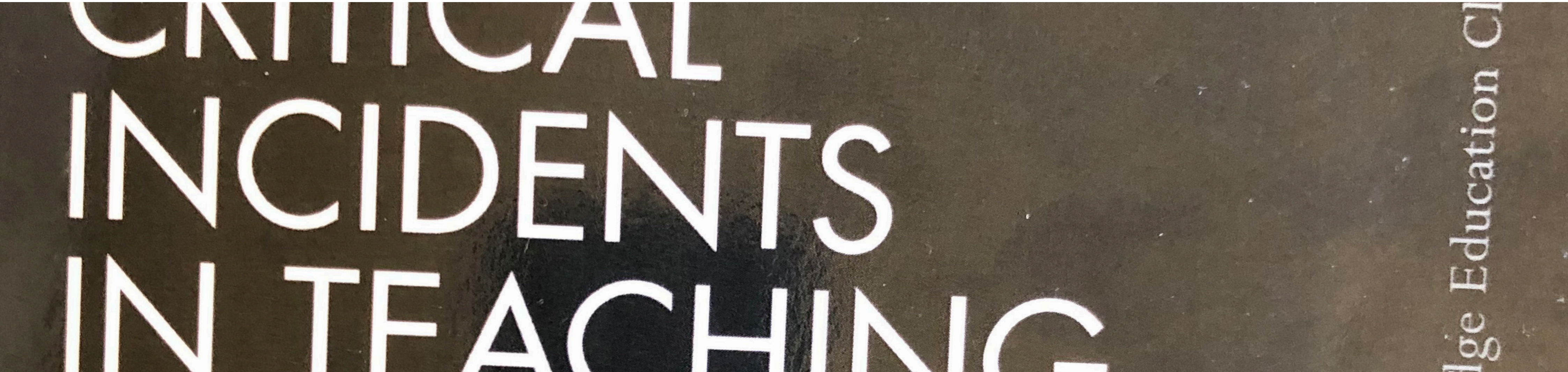

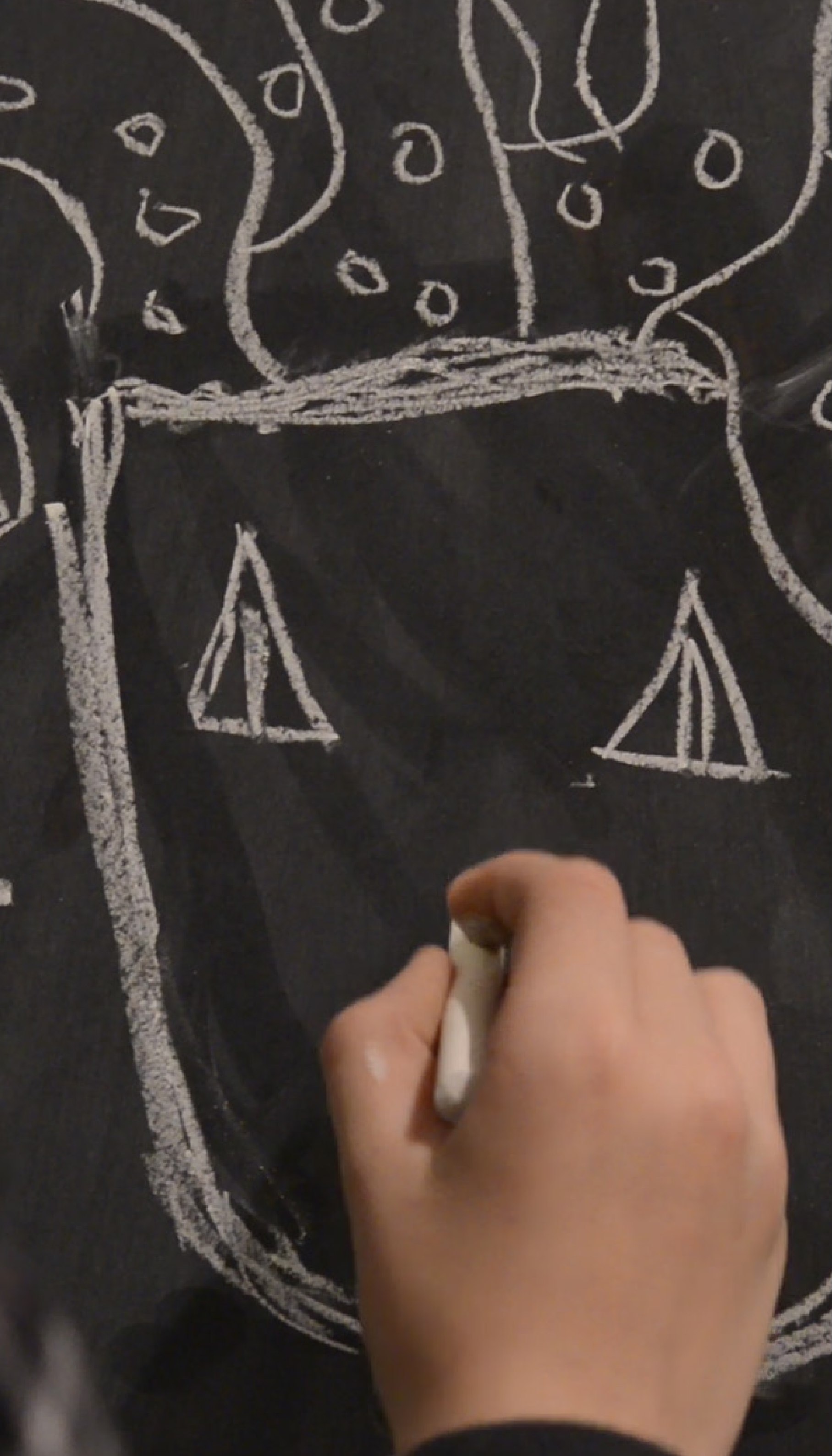

8. BETWEEN PROCESS AND PRODUCT: PERSPECTIVES AND REFLECTIONS

Dialoghi in piazza is a wide-ranging project still in progress.

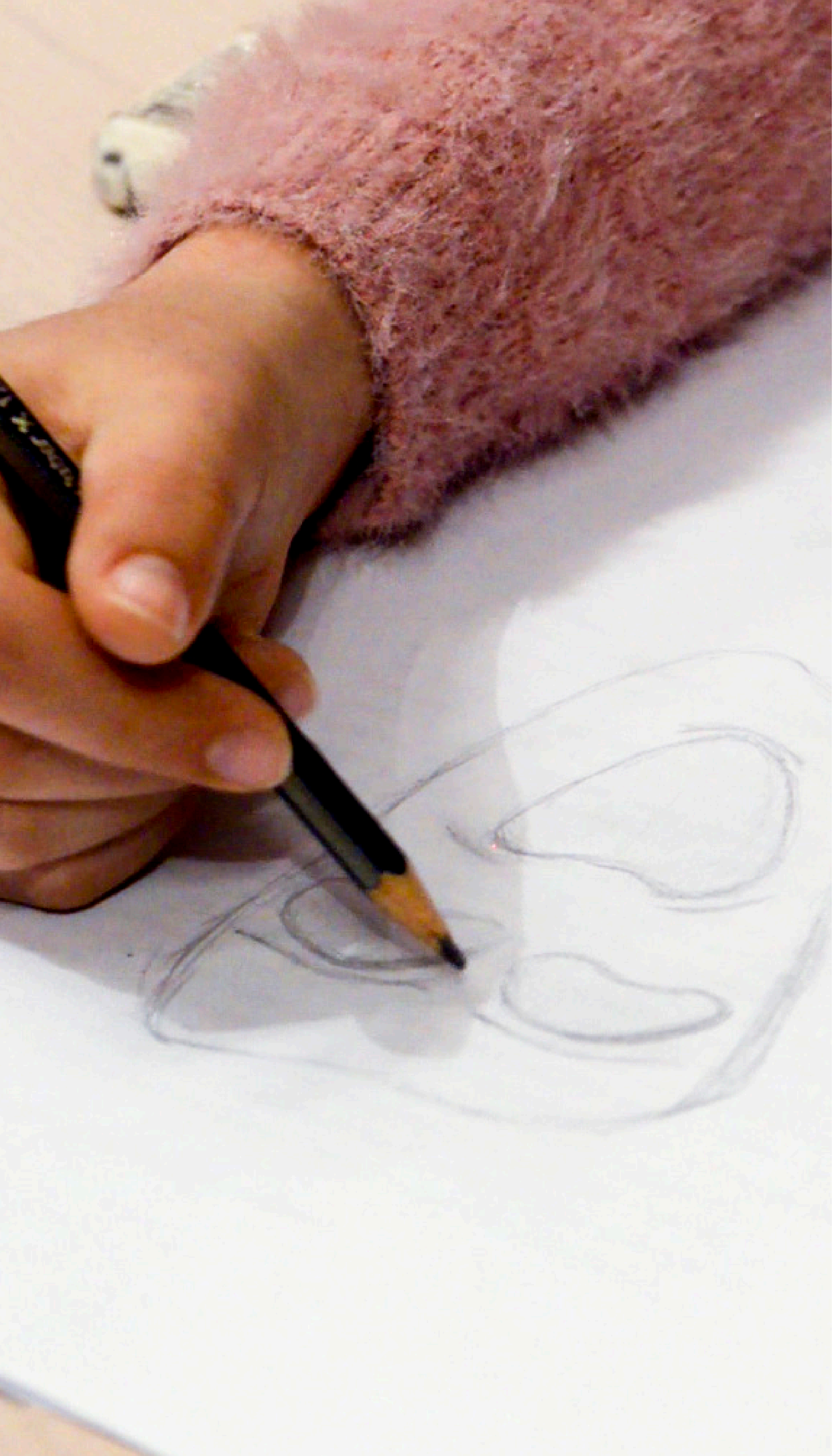

Stimulating active participation in the improvement of the surrounding environment is a primary objective of education for global citizenship and sustainability, an important link between the school and the community (ASVIS, 2018)

However, creating and making changes in urban spaces is extremely complex.

Dialogues in the square is currently a large laboratory, in which talents and abilities emerge in teachers and students, but it does not lack challenges.

SOME OPEN ISSUES

• THINGS TAKE TIME, HOW LONG WILL IT TAKE TO SEE A TANGIBLE RESULT?

Between observations, consultations, authorizations, an action project on urban spaces requires considerable time and the ability to “stay” for a long time on obstacles to overcome, apparently unanswered questions, unexpected effects (Lorenzoni, 2019).

• DEVELOPMENT OF THE PATH: FOLLOWING THE LOGIC OF AN “A PRIORI” PROJECT DESIGN OR WELCOMING THE UNEXPECTED?

It is difficult to rely on an exclusively rational design: the most significant developments derive from meetings, opportunities to be seized, unexpected events. Re-design is continuous and requires challenging coordination.

• COMMUNICATE TO SHARE: WHICH INSTRUMENTS?

In a project on urban spaces, analyses, complaints and proposals for action must be shared. Working with communication tools is essential: skills that are usually developed in school are necessary to produce effective messages. However, they can enter the school through the interaction with experts.

• STUDENTS' SENSE OF SELF-EFFICACY: EASY FOR THE PROCESSES, BUT THE PRODUCTS

Students can be easily motivated, they show commitment and interest, often involving their parents directly. However, they have an idea of a very concrete and immediate result: they would like to translate the processes into actions and products at a rate that is incompatible with long-term objectives. The unravelling of the path risks creating a sense of discouragement and ineffectiveness of the students' action.

CRUCIAL INSTRUMENTS AND STRENGHTS

• the creation of a group involving classes and teachers of different levels with a sense of belonging to the project and the desire to plan together.

• the meticulous and co-constructed work carried out, favouring moments of active and inter-generational exchange in extra-curricular contexts and accustoming the students to really delve into problems.

• the development of ever wider synergies with institutions and local players interested in the identified objectives.

• the relationship with artists and experts who interact continuously with teachers and students, appreciate and value their ideas and collaborate to give effective form to communication.

• the possibility of making use of work spaces outside the school that were rich of suggestions, such as those made available by Macro Asilo, which make it possible to broaden and multiply the creative process, making it concrete and "public" and creating intermediate products (blackboard).

• joining the meta-project of the Third Paradise and the Pistoletto's Rebirth Forum which created a new creative scenario and allowed for new relationships to be developed.

• the construction of a framework for the observation, monitoring and evaluation through tools developed with the Chair of Psychology of Education and E-learning of the University of Bari. This will allow to understand the pedagogical and cognitive potential of the experience.

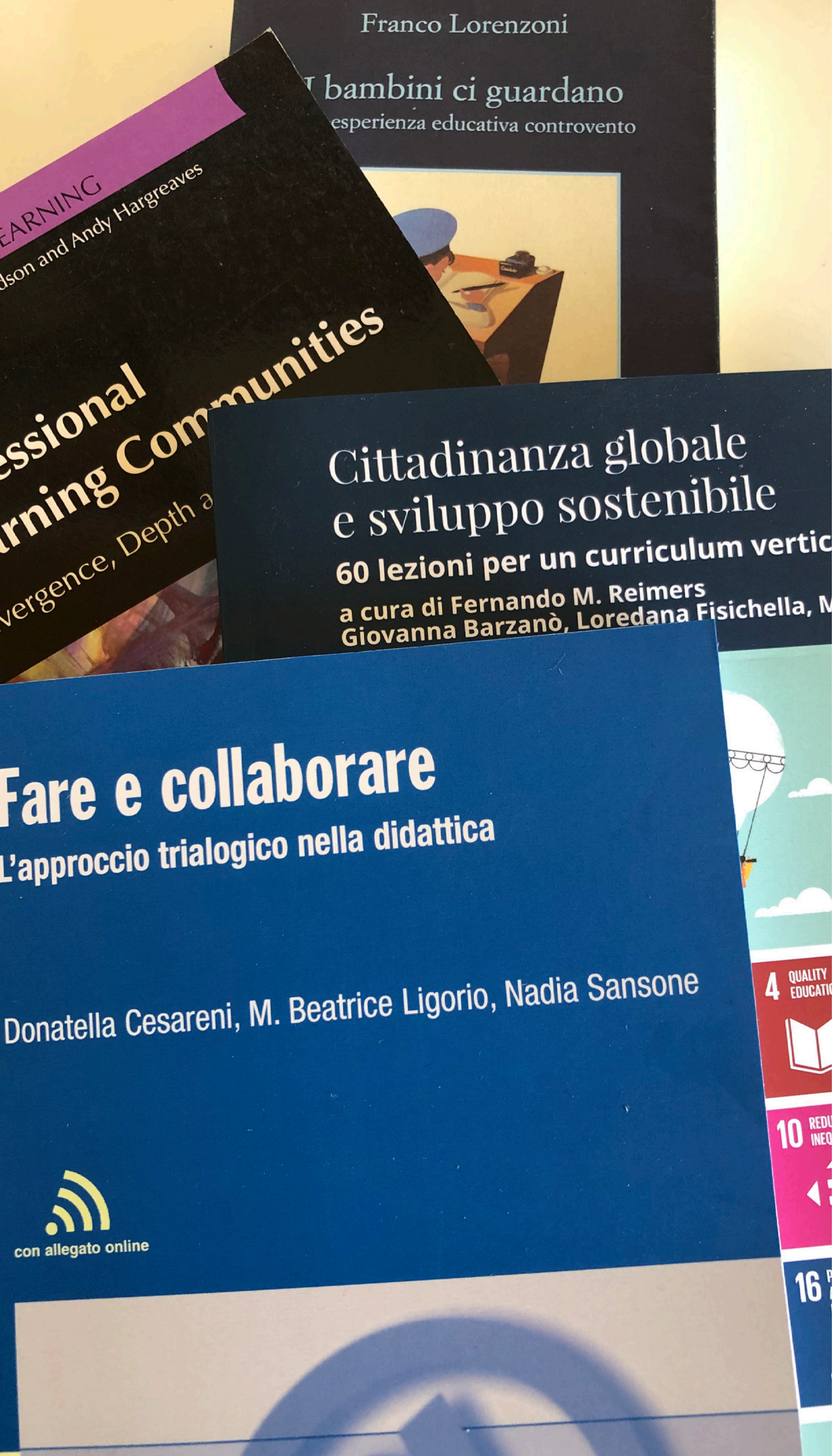

9. REFERENCES

Anderson, G. (2017). Participatory action research (PAR) as democratic disruption: new public management and educational research in schools and universities. International Journal of Qualitative Studies in Education, 30(5), 432-449.

ASVIS, (2018) Strategia Italiana per l'educazione alla cittadinanza globale, Provincia Autonoma di Trento

Banks, J. A. (2004). Teaching for Social Justice, Diversity, and Citizenship in a Global World. The Educational Forum, 68(4), 296-305.

Barzanò, G., & Zacchilli, E. (2017). Cittadinanza europea e cittadinanza globale: tra appartenenze e valori. In P. Corbucci, & M. Freddano, Cittadinanza europea. Torino: Loescher.

Barzanò, G., Cortiana, P., Jamison, I., Lissoni, M., & Raffio, L. (2017). New means and new meanings for multicultural education in a global—Italian context. Multicultural Education Review, 9(3).

Barzanò, G., Raffio, L., Lissoni, M., & Mallardi, R. (2017, in press). Engaging in intercultural and interreligious dialogue at school: hints from Rete Dialogues and the Generation Global project. Scuola Democratica.

Cesareni,D., Ligorio, M.B., Sansone, N., Fare e collaborare,(2018) , Milano, Angeli

Fielding, M. (2011). Radical democratic education and emancipatory social pedagogy: prolegomena to a dialogue. In C. Cameron, & P. Moss, Social Pedagogy and Working with Children: Engaging with Children in Care (pp. 177-194). Jessica Kingsley Publishers..

Freire, P., & Macedo, D. (1995). A dialogue: Culture, language, and race. Harvard Educational Review, 65, 377—403.

Gee, J. (2017). Identity and diversity in today's world. Multicultural Education Review, 9(2), 83-92.

Greenhow, C., & Lewin, C. (2016). Social media and education: reconceptualizing the boundaries of formal and informal learning. Learning, Media and Technology, 41(1), 6-30.

Ligorio, M. (2010). Dialogical relationship between identity and learning. Culture & Psychology, 16, 109-115.

Lorenzoni, F.(2019) I bambini ci guardano, Palermo, Sellerio

Paavola, S., & Hakkarainen, K. (2005). The Knowledge Creation Metaphor – An Emergent Epistemological Approach to Learning. Science & Education, 14, 535—557.

Riley, K. (2017). Re-Creating Schools as Places of Belonging: The Art of Possibilities. Retrieved from The Staff College: http://thestaffcollege.uk/publications/re-creating-schools-as-places-of-belonging-the-art-of-possibilities/

Rizvi, F. (2009). Towards cosmopolitan learning. Discourse: Studies in the Cultural Politics of Education, 30(3), 253-268.

Roxas, K., Cho, J., Rios, F., Jaime, A., & Becker, K. (2015). Critical cosmopolitan multicultural education (CCME). Multicultural Education Review, 7(4), 230-248.

Schereens, J. (2009). Informal Learning of Active Citizenship at School. Dordrecht: Springer.

Stoll, L., & Louis, K. S. (2008). Professional learning communities: Divergence, depth and dilemmas. Maidenhead: Open University Press/McGraw-Hill Education.

Tripp, D. (1993-2012) Critical incidents in teaching, London , Routledge

UNESCO. (2016). The ABCs of Global Citizenship Education. Retrieved from http://unesdoc.unesco.org/images/0024/002482/248232e.pdf

Woods, P. (2012). Critical Events in Teaching & Learning. Routledge
